# Opto-fluidically multiplexed assembly and micro-robotics

**DOI:** 10.1038/s41377-024-01406-4

**Published:** 2024-02-27

**Authors:** Elena Erben, Weida Liao, Antonio Minopoli, Nicola Maghelli, Eric Lauga, Moritz Kreysing

**Affiliations:** 1 Max Planck Institute of Molecular Cell Biology and Genetics, Dresden, 01307 Germany; 2 Center for Systems Biology, Dresden, 01307 Germany; 3 Institute of Biological and Chemical Systems – Biological Information Processing. Karlsruhe Institute of Technology (KIT), Eggenstein-Leopoldshafen, 76344 Germany; 4 Department of Applied Mathematics and Theoretical Physics, University of Cambridge, Cambridge, CB3 0WA UK

**Keywords:** Optical manipulation and tweezers, Optofluidics

## Abstract

Techniques for high-definition micromanipulations, such as optical tweezers, hold substantial interest across a wide range of disciplines. However, their applicability remains constrained by material properties and laser exposure. And while microfluidic manipulations have been suggested as an alternative, their inherent capabilities are limited and further hindered by practical challenges of implementation and control. Here we show that the iterative application of laser-induced, localized flow fields can be used for the relative positioning of multiple micro-particles, irrespectively of their material properties. Compared to the standing theoretical proposal, our method keeps particles mobile, and we show that their precision manipulation is non-linearly accelerated via the multiplexing of temperature stimuli below the heat diffusion limit. The resulting flow fields are topologically rich and mathematically predictable. They represent unprecedented microfluidic control capabilities that are illustrated by the actuation of humanoid micro-robots with up to 30 degrees of freedom, whose motions are sufficiently well-defined to reliably communicate personal characteristics such as gender, happiness and nervousness. Our results constitute high-definition micro-fluidic manipulations with transformative potential for assembly, micro-manufacturing, the life sciences, robotics and opto-hydraulically actuated micro-factories.

## Abstract (web summary)

Feedback control of optically generated flows enables precise, dynamic particle assembly and complex micro-robotics.

## Graphical abstract

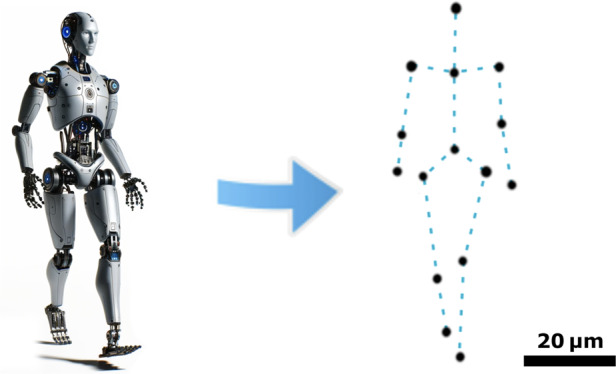

## Introduction

Precise manipulation of microscopic particles in solution remains an important area of research, with applications in life sciences, biomedical research, nanoscience, engineering, and physical research^1–3^. Contemporary approaches to micromanipulation include optical tweezers^4–6^, magnetic robots^7^, optically actuated micro-steerers^8,9^, micro-rockets^10^, and microtools^11^, as well as Marangoni streamers^12^ and Janus particles^13^.

These and other techniques, as detailed in Supplementary Table 1, typically require specific material properties of the particles or supplementary probes, and necessitate high-intensity laser exposure, or lack the optical control capabilities to multiplex and manipulate multiple particles.

Beyond these established methods for micromanipulation, microfluidics in multi-pump actuated microfluidic chambers has long been suggested as a solution for the precision handling of micron-sized particles^14–16^ and the assembly of complex structures^15,17,18^.

While theoretically sound and experimentally demonstrated for up to three particles^19^, the complexity of the suggested approaches scales unfavorably with particle number, with six being the suggested upper limit^15^. And even before this limit is reached, this non-localized character of flow fields actuated by micro-fluidic pumps imposes severe experimental challenges, such as high velocities distant to regions of interest, as well as an unfavorable scaling of hardware requirements with particle numbers.

The physics of flow fields in pump-actuated chambers severely limit the potential of microfluidics for object handling, thereby rendering the development of hydraulically actuated micro-robots with degrees of freedom comparable to those in contemporary macro-scale robots elusive.

Here we propose an alternative, opto-fluidic approach to enable precision micromanipulation of particles. This overcomes previous limitations, does not require any hardware changes to transition from single to many particle manipulations, shows an unexpected speed-up upon multiplexing, and owes itself to realize analytically tractable high-definition robotics with large numbers of degrees of freedom.

## Results

### Iterative positioning of particles, one at a time

**Fig. 1:**
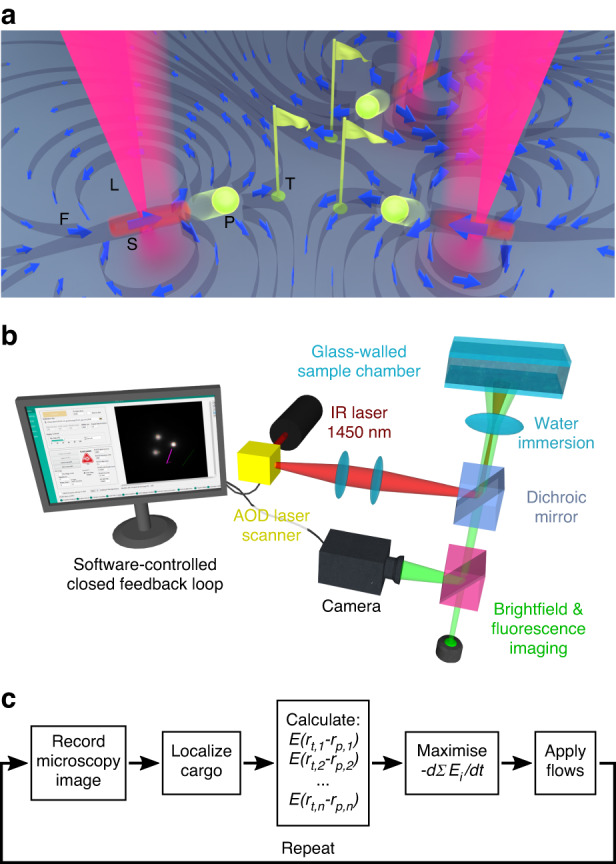
Concept of multi-particle positioning via feedback-controlled multiplexed thermoviscous flows. **a** Topologically rich, laminar thermoviscous flow fields (F) for the simultaneous transport of micro-particles (P) to specific target locations (T) are generated by rapidly scanning mildly heating infrared laser beams (L) along suitable, multiplexed scan paths (S). **b** Laser position in the sample is controlled by a 2-axis acousto-optic deflector, which allows sampling frequencies of up to a few kilohertz. The deflector is connected to a LabVIEW-based control software that also receives camera images of the sample, enabling image-based feedback control of the flows. **c** Generalized feedback loop scheme to facilitate the transport of cargo in a thin fluid chamber via thermoviscous flows. *E* is a freely selectable variable of the system that should be minimized (or maximized) by the feedback control. In this paper, we chose *E* to be the sum of distances of all particles to their targets. ***r***_*p,n*_ is the position of the *n*th particle and ***r***_*t,n*_ is the position of the *n*th target

We make use of optically generated thermoviscous flows^20,21^, which we have previously shown can be used to move and confine individual colloidal particles^22,23^. In brief, these laminar flows arise from the complex interplay between thermal expansion and viscosity changes with temperature, making them a second order physical effect O(*ΔT*^2^). These directed flows can be optically induced at any position in the fluid via the directed spatial scanning of a weakly heating, single mode infrared laser beam (${\lambda}$ = 1455 nm, Fig. 1a) at low kilohertz repetition frequencies using standard acousto-optical modulators with a high-definition fluorescence microscope (Fig. 1b). It has been shown analytically that under two-dimensional confinement the induced flow field is strongly localized near the laser scan path and decays as 1/*r*^2^ in the far field^24^. We complement these laser scanning and imaging optics with an extended closed-loop feedback scheme (Fig. 1c) to sequentially reduce particle positioning errors with time.

**Fig. 2:**
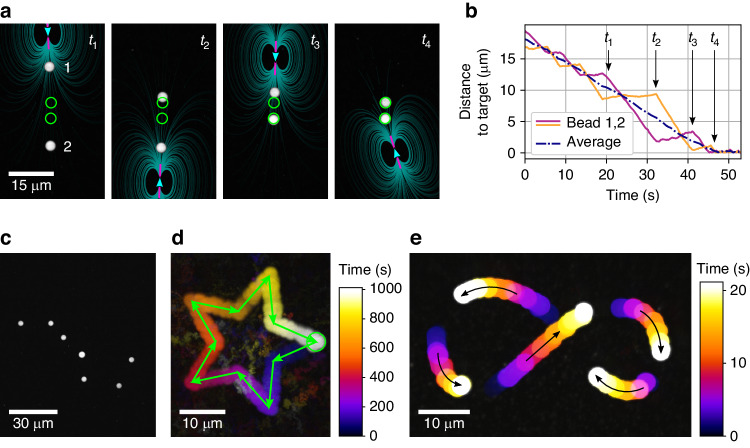
Iterative and dynamic alignment of multiple particles via the sequential application of single thermoviscous flow fields, with sub-micrometer accuracy and dynamic alignment via mobile destinations. **a** Basic sequential iterative alignment of two particles via optically generated flow fields (see also Supplementary Video 1) and (**b**) analysis of temporal convergence. **c** Alignment of multiple micro-particles to form the *“big” dipper* via the iterative scanning of a single laser beam. **d** Guidance of a single particle along a star-shaped path via moving the destination of positioning (see also Supplementary Video 3). The particle follows the path with a precision of 288 nm (see Fig. S1). **e** Combining the iterative alignment of multiple particles with moving destinations to guide particles along a self-intersecting trajectory

In the following, we demonstrate that due to the local character of flows, already a sequential alignment scheme (Fig. 1c) allows us to position multiple particles iteratively with time. We firstly demonstrate this for two 3 µm-sized polystyrene particles to be positioned only one diameter apart, by pushing always the particle with the largest individual error, in this case the largest individual distance from its target (Fig. 2a, Supplementary Videos 1 and 2 and Fig. S8). Contrasting classic microfluidic approaches, we find that positioning of two particles close to each other at pre-assigned destinations shows only a modest hydrodynamic coupling, and with a net error of displacement that decreases nearly linearly with time (Fig. 2b), while circumventing problems of large flow velocities in undesired positions, a problem associated with previous suggested microfluidic assembly approaches^15^.

Due to the localized character of the optically induced flow field, our approach can easily be employed to position even more particles in order to assemble quasi-static structures on the micron-scale (Fig. 2c), readily exceeding practical and theoretical limits for particle numbers to be handled in pump-actuated chambers.

The approach not only allows control of end point positions, but also the controlled guidance of particles with nano-scale precision using dynamic set-points (Fig. 2d, Supplementary Video 3 and Fig. S1), or the movement of multiple particles along complex paths, including self-intersecting trajectories (Fig. 2e) that would not be accessible by any steady flow field or classic microfluidics.

In contrast to previously proposed pump-based microfluidic approaches^15^, our approach does not require the relative immobilization of particles to artificially reduce the number of degrees of freedom in the system. This enables the precise relative alignment of particles at considerable distances. The size of particles that can be controlled ranges from hundreds of nanometers to tens of micrometers (Fig. S5). Our approach can manipulate particles regardless of their material properties (Fig. S7) and in both high and low-viscosity fluids (Fig. S6).

### Multiplexing and non-linear acceleration

While our approach already facilitates multiparticle assembly via the sequential iterative application of flow fields to position individual particles, we find that the optical flow field generation can readily be multiplexed, leading to an unexpected, non-linear acceleration of complex manipulations.

**Fig. 3:**
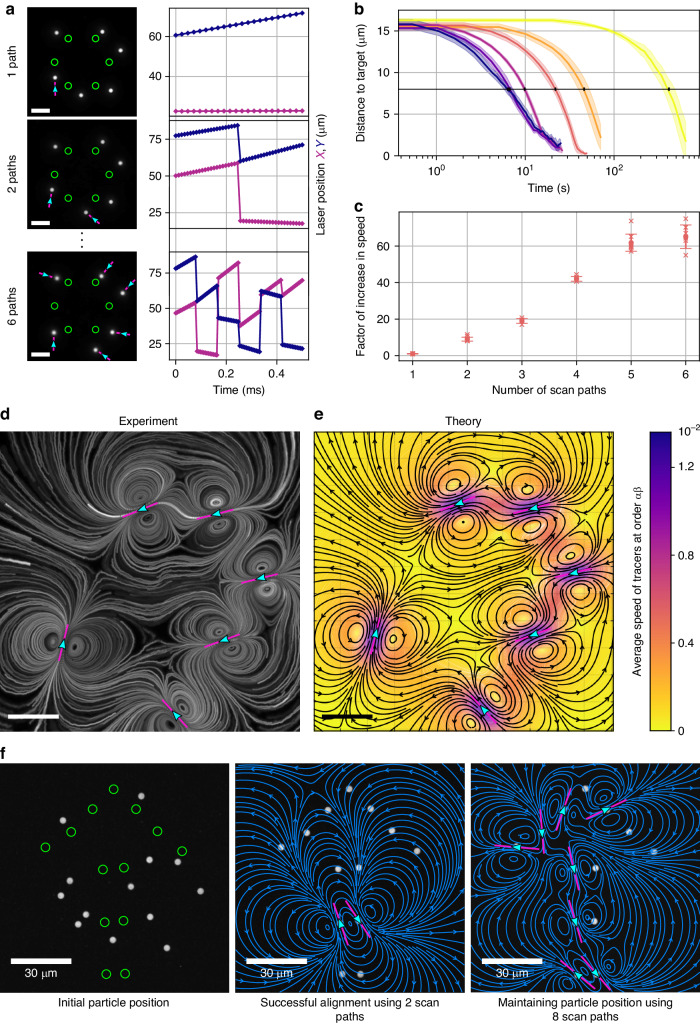
Non-linear acceleration of alignment processes via the time-shared optical induction of flow fields. **a** Flow multiplexing by time-shared scanning of laser paths on time scales below the thermal relaxation time, while keeping the average heating per path constant (Fig. S4). For this, the scan period, which corresponds to the thermal relaxation time, is sub-divided to facilitate multiple scan paths, while the laser power is increased proportionally to the degree of multiplexing (Fig. S2). See also Supplementary Video 4 for a dynamic visualization of this concept. Scale bars: 15 µm. **b** Analysis of distance between particles and their destination and (**c**) convergence speed for up to 6 simultaneously applied flow fields, reveal a highly non-linear speedup, with 6 scan lines yielding a (65 ± 6)-fold acceleration over the single scan path. Curves shown in (**b**) represent the distance over time, averaged over all 6 particles and over 5 to 8 repetitions each; the shaded area displays the standard deviation over the repetitions. The speed in **c** is calculated as the inverse of the convergence time, which is measured as the time at which the average (over all 6 particles) distance to the targets is approximately halved (reduced to 8 µm, see black horizontal line in (**b**). Again, the average of 5 to 8 repetitions is shown, the error bars represent the standard deviation. **d** Visualization of a typical, topologically rich flow field as it occurs for 6-fold multiplexing. Scale bar: 15 µm. **e** The corresponding analytical result building on a recently published mathematical model for a single scan path^24^, showing excellent agreement with the experiment and reproducing all 22 topological defects in the flow field. Scale bar: 15 µm. **f** Parallel assembly of a humanoid robot from randomly dispersed beads: The assembly can be achieved both with low (2, see Supplementary Video 5) and high degree of flow-multiplexing (8)

Inspired by the time-sharing in optical traps^4^, we decided to multiplex the generation of laser scan paths below the diffusion-limited thermal relaxation time of the chamber (Fig. 3, Figs. S2–4 and Supplementary Video 4).

Unlike optical tweezers, where an increase in laser power proportional to the number of traps serves the multiplexing of identical traps, we found that in our approach the velocity for each individual flow field even increases upon multiplexing (Fig. S3). As a net result of parallelization and individual speed-up, we observed a highly non-linear reduction in assembly time, when repeating the same task (Fig. 3a–c and Supplementary Video 4). Specifically, we observed an about (65 ± 6)-fold speed-up when using 6 scan paths rather than 1 to arrange particles into a hexagon shape (Fig. 3c).

This unexpected speed-up is possible due to the physical nature of thermoviscous flows, which scale with repetition rate^21^, rather than laser beam velocity, which enables the multiplexed optical actuation of several flow fields during each heat diffusion limited scan period (Fig. 3d).

To show that beyond the speed-up multiplexed flow fields yield mathematically predictable results, we built on a recently developed analytical model for thermoviscous flows for a single laser scan path^24^. On this basis, we computed the time-averaged velocity of tracer particles in the fluid resulting from scan patterns with multiple scan paths, in the limit of small density changes and viscosity changes induced by the laser heating of the fluid. To leading order, this is proportional to the sum of the net displacement of tracers due to each individual scan path.

When comparing experimental results and theory for a complex flow field generated by 6 multiplexed laser scan paths, we found excellent agreement in the presence of as much as 22 topological defects (Fig. 3d, e).

This quantitative modelling furthermore illustrates that, by inducing flows locally, our approach overcomes the problem of requiring high flow velocities, which are experimentally non-feasible, far away from the particles in order to induce moderate flow velocities near them, thereby mitigating historic application limitations^15,17,19^.

Furthermore, and in contrast to previous suggestions based on pump-actuated systems, our all-optical approach maintains a constant level of hardware requirements regardless of the number of particles being manipulated.

Therefore, leveraging the dynamic optical localization of flow fields, we can readily assemble mimics of humanoid robots via dynamic series of complex and topologically rich flow fields that transport or stabilize particle positions in the presence of diffusion (Fig. 3f and Supplementary Video 5).

### Context-aware strategies to enhance convergence, stability and precision of dynamic micro-robots

State-of-the-art robots now display numerous degrees of freedom, dynamic motion, and high precision. In fact, humanoid robots have advanced to the point where they can exhibit sophisticated motion and replicate emotional body language^25–27^. Our method enables the relative positioning of larger numbers of particles in relation to one another. As these do not need to be immobilized in their relative positions^15^ in order to reduce the number of degrees of freedom, particles remain mobile at all times, which is a pre-requisite for their dynamic actuation.

While complex macroscopic robots need to be conditioned to avoid self-collisions (e.g., not to stumble over their own feet), we found a similar and in parts more difficult problem that requires attention in our microscopic assemblies. That is, particles that get very close to each other, may show significant, but resolvable, crosstalk, not only due to steric hinderance, but due to unspecific hydrodynamic coupling of flows and particles at small distances.

Yet, additional flexibility is induced, when transitioning from flow fields that match particle positions with regions with highest velocities, to fields that are slightly displaced, consequently combining high flow speeds with significant gradients in velocity.

**Fig. 4:**
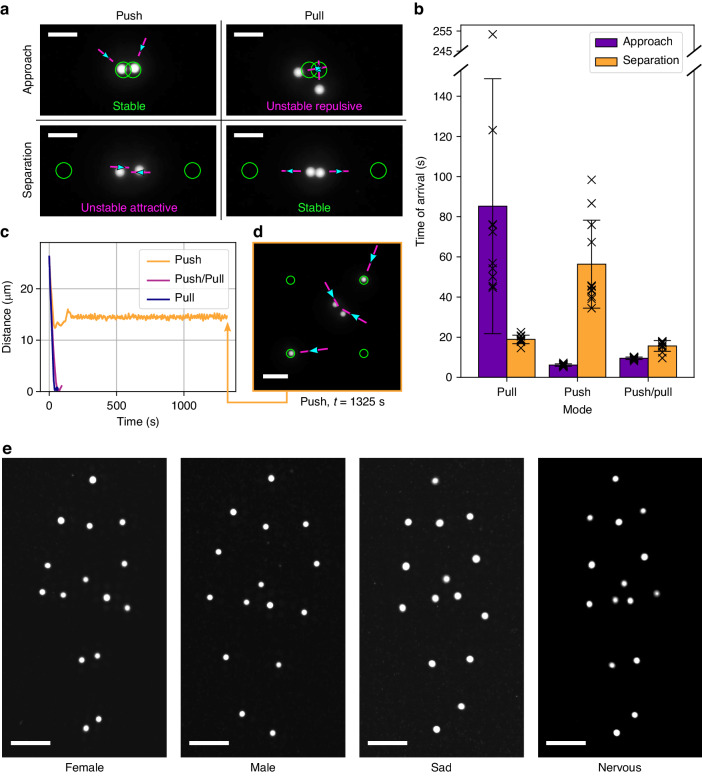
Humanoid robots capable of expressing emotional body language, facilitated by context-aware dynamic alignment strategies to minimize hydrodynamic coupling. **a** In close proximity of particles (separation in the range of the particles’ size), hydrodynamic coupling may occur and impact the assembly. This can be resolved via context-aware decisions to switch from pushing to pulling. We observe approach and separation to be stable via pushing and pulling fields respectively, while both the separation via pushing flow fields, and the approach via pulling fields yield unstable repulsive and unstable attractive convergence dynamics, respectively (see also Supplementary Video 6). Scale bars: 10 µm. **b** Statistics of convergence dynamics showing context-aware decisions (‘push or pull’) to be most efficient and reliable. Time of arrival is defined as the time from the start of the alignment until the average distance of both particles to their targets is decreased to 2 µm. In the bar plot the time of arrival is averaged over 10 or 11 repetitions each, error bars show the standard deviation. **c**, **d** Context-aware decisions might also prevent oscillatory stalling behaviors in confined spaces (see Supplementary Video 7). Scale bar: 15 µm. **e** Humanoid micro-robots can be assembled and animated sufficiently accurate to express emotional body language (see Supplementary Videos 8 and 9). Differences in perceived gender or emotion are caused by changes in the body anatomy (female vs. male) and by subtle changes of the dynamic motion patterns of the figures^28^ (e.g., stiffer body movement for nervous vs sad, compare video). Scale bars: 15 µm

In particular we found that while particles might be readily brought together and held in close proximity by ‘pushing’ flow fields, their efficient separation during which particles remain stably on individual paths, strongly benefits from ‘pulling fields’ (Fig. 4a and Supplementary Video 6). Pushing flows result in an attractive instability during separation attempts, while, conversely, pulling flow fields result in a repulsive instability, and large fluctuations of convergence times (Fig. 4b). Context-aware local decisions to pull or push, always showed fast convergence (Fig. 4b), and reduce the risk of oscillatory behavior in confined arrangements (Fig. 4c, d and Supplementary Video 7), already by taking the position of the next neighbor into account (see methods section and Supplementary Video 10 for decision model).

Drawing inspiration from humanoid robotics research and studies on the perception of motion patterns^28^, we finally animated walking humanoid robots at about 10^−15^ times the weight of their human-scale hydraulically actuated counterparts by using hundreds of multiplexed and continuously changing optically and contextually generated flow fields to actuate the individual ‘body parts’.

Displaying up to 30 degrees of freedom, their dynamics are sufficiently well-defined to reliably communicate human attributes such as gender, happiness or even nervousness via their body language, as displayed while walking (Fig. 4e and Supplementary Videos 8 and 9).

## Discussion

We introduced a novel approach to facilitate parallel hydrodynamic manipulations of microparticles, which leverages optical control to generate topologically rich, dynamic flow fields, exhibiting a nonlinear speedup upon multiplexing.

Compared to proposals to use pump-actuated microfluidics in multi-inlet chambers, our approach is easy to implement and control, all-optically through a microscope, and is not restricted to low particle numbers. Explicitly, we have shown that we can precisely control the position and motion of 15 microparticles independently and in a parallel fashion which constitutes control over 30 degrees of freedom. Furthermore, we believe that at this number of particles our technique is not limited by the physics of the system, rather by current limitations in the capability of the control algorithm and its processing speed. With rapid implementation and advanced control, it might be feasible to position one particle every few micrometers, which is the typical length scale over which the flow fields exhibit significant decay^22,24^. We predict that this will facilitate parallel control of larger numbers of particles with even smaller distances in between.

Unlike complementary approaches such as optical tweezers^4–6^, magnetic robots^7^, optically actuated micro-steerers^8,9^, Marangoni streamers^12^ or Janus particles^13^, that necessitate specific material properties of particles or supplementary probes, our approach is contact- and laser exposure-free, which renders it attractive for the use with cells and developing embryos^29,30^. Moreover, this technique is inherently usable with any objects that can be transported by flows. As we do not depend on the mechanical interfacing with a probe, the hydrodynamic approach effectively also circumvents challenges related to dexterity in handling objects known from macro-scale robotics.

The laminar nature of the flows ensures that, despite the high complexity of the flow fields, manipulations are straightforward, as demonstrated by the sufficiency of simple control algorithms. In the future, adopting more advanced control algorithms such as proportional–integral–derivative (PID) control, model predictive control (MPC, enabled through a full analytical model of the flows^24^) or reinforcement learning should allow us to solve generalized problems such as manipulating particles in the presence of external flows and positioning of even higher numbers of closely spaced particles. This, combined with advanced light field synthesis techniques, will further expand the range of applications and practical utility. Specifically, we see opportunities in three areas: i) the field of biology and the life sciences, where our technique could help study cell-cell interactions or the effects of spatial arrangement on tissue formation. For instance, generating spatially well-defined precursor colonies for forming organoids could address the problems of poor reproducibility and high variability currently faced in the field^31^. ii) In the field of biochemistry, RNA-protein condensates have emerged as a crucial aspect of cytoplasmic organization over the last decade^32,33^. The advanced manipulation techniques introduced here could be extended to manipulate these condensates in vivo, or in reconstituted in vitro analogs, to assess their physicochemical properties and interactions. Furthermore, in the field of iii) engineering and manufacturing, our capabilities to handle objects irrespective of their material properties might allow the operation of light-controlled micro-factories where materials are positioned strategically and timely^18,34^.

## Materials and methods

### Optical setup for the generation of thermoviscous flows

The opto-fluidic setup used (in large parts previously described by Mittasch et al. ^29^) consists of, (a) an infrared laser scanning unit and (b) an inverted microscope for laser projection and fluorescence imaging. The infrared unit employs a fiber-based Raman laser (continuous-wave mode, 20 W maximum power, CRFL-20-1455-OM1, Keopsys) at a wavelength of 1455 nm, a LabVIEW^35^-controlled 2D acousto-optic deflector (AOD, AA.DTSXY-A6-145, Pegasus Optik) for rapid scanning and multiplexing, and a 4-f telescope (lenses AC254-C series, Thorlabs) to relay the scanner into the microscope’s back focal plane.

Equipped with bright-field and fluorescence imaging optics, the microscope (IX83, Olympus) uses an infrared-coated objective lens (60× UPLSAPO, NA = 1.2, W-IR coating, Olympus) used with heavy water (D_2_O) for simultaneous imaging and scanning, and a dichroic mirror (F73-705, AHF) to combine the two optical paths. A bright-field illumination is provided by a high-power LED (M565L3, Thorlabs). Confocal fluorescence imaging is facilitated by a VisiScope system (Visitron), Yokogawa CSU-X1-A12 scan head, and sCMOS camera (Zyla 5.5, Andor).

### Construction of chambers with probe particles for precise positioning

The chambers consist of a liquid film with immersed particles, sealed between a cover slide and cover glass using dental impression material (Identium® Light, Kettenbach Dental). The chamber contains a probe liquid mixed with smaller fluorescent polystyrene (PS) beads as probe particles and larger polystyrene beads to define the chamber thickness (‘spacer beads’).

For positioning experiments a 50 to 60% (v/v) glycerol (puriss., 15523-M, Sigma-Aldrich)-water mixture was used as a probe liquid. The probe liquid also contained 0.2 to 0.3% (v/v) Tween® 80 (P1754, Sigma-Aldrich) to prevent adhesion of probe particles to chamber walls.

In most positioning experiments, fluorescent PS beads with a 3 µm diameter (Fluoresbrite® YG Microspheres, mean diameter 3.06 μm; 17155-2, Polysciences) were used as probe particles and 5 µm non-fluorescent PS beads (non-functionalized polystyrene, mean diameter 5.15 μm; PS06N, Bangs Laboratories, Inc.) or 5 µm fluorescent beads based on melamine resin (75908-10ML-F, Sigma-Aldrich) as spacers, yielding approx. 5 µm thick chambers. For positioning experiments requiring smaller probe particles (e.g., the humanoid robots in Fig. 4e), the chambers contained 1.6 µm fluorescent PS beads (Encapsulated Magnetic Polymer-COOH, mean diameter 1.63 μm; ME04F, Bangs Laboratories, Inc.) as probe particles and 2 µm fluorescent PS (Fluoresbrite® YG Carboxylate Microspheres, mean diameter 2.1 µm, 09847-5, Polysciences) beads as spacers, yielding approx. 2 µm thick chambers.

The chamber thickness was additionally defined by the exact volume of liquid placed between the glass planes (2 µL for 5 µm high chambers and 0.8 µL for 2 µm high chambers). 0.1 µm diameter fluorescent PS particles (Fluorescent Carboxyl Polymer Particles, mean diameter 0.11 μm, FC02F, Bangs Laboratories, Inc.) were used to identify the background and subsequently measure the chamber height in a *z*-stack.

To visualize the flow fields generated by one or multiple scan paths (Fig. 3d and Fig. S3), chambers filled with a highly viscous liquid (undiluted glycerol in Fig. 3d and honey in Fig. S3) and 0.5 µm large fluorescent PS particles (Fluorescent Polystyrene, mean diameter 0.52 μm, FS03F, Bangs Laboratories, Inc.) were used.

The construction protocol and characteristics of the chambers were previously described in large parts by Erben et al.^22^.

### Feedback loop and alignment algorithm based on maximum distance to target

In this section we describe the feedback loop used for the positioning of multiple particles. See also Fig. S8 for a visualization of the essential parts of the feedback loop as described here.

Upon every execution of the feedback loop, first, a microscopy image of the sample is recorded in a custom-coded LabVIEW software. Then particles are localized in the image using a standard routine utilizing thresholding segmentation followed by center of mass calculation. The current particle coordinates are transferred to a Python program via Transmission Control Protocol (TCP). In the Python code the particles are first assigned to the target destinations using the Hungarian algorithm with the particle displacements from the targets as the cost^36,37^. For this we used the munkres-package for Python by Brian Clapper^38^. Then the particle with the largest distance is selected and a laser scan path is calculated for this particle according to the following rules: (i) The laser scan path lies on the line connecting the particle and its associated target. (ii) The laser scan path has a defined length and is placed at a defined distance from the particle. Distance and length can be chosen freely, but are usually kept constant over the course of the positioning. (iii) The laser scan path is set such that the flows will be either ‘pushing’ or ‘pulling’ the particle. When ‘pushing’, the laser scan path is positioned on the side of the particle that is opposite to the target, while for ‘pulling’ it is positioned on the side of the target. In both modes the laser scanning is directed away from the target, such that the flows are directed towards the target, resulting in the particle moving toward its associated target both in the ‘pushing’ and in the ‘pulling’ case. We previously showed that positioning a single particle using pulling flows results in the equivalent positioning characteristics as for pushing flows^22^. Here we use pushing flows by default, but pulling flows are used for context-aware decisions (see below). In the case of scan path multiplexing that we introduced in Fig. 3, *N* laser scan paths are calculated for the *N* particles that have the largest distance to their associated targets, where *N* is the degree of multiplexing. As described already in the main text, the regular scan period is then equally shared between the *N* scan paths. After calculating the laser scan path(s), its coordinates are sent back to LabVIEW, where the laser scanning is then executed. This loop is repeated at frequencies of typically 10 Hz. For dynamic positioning of particles, the target destinations are additionally updated automatically in a separate loop, that repeats at the same frequency or lower.

Recording of the microscopy images, particle localization and application of the laser scanning are carried out in LabVIEW, the remaining steps in Python. The software was developed by Nicola Maghelli and Sergei Klykov at MPI-CBG and further optimized commercially by JKI (US-based company).

### Simulations of thermoviscous flows

To compute the trajectories of tracer particles (Fig. 3e, f and Supplementary Videos 8 and 9) and their time-averaged velocity (Fig. 3e) theoretically, we use our recent work characterizing the average flow field due to an individual scan path derived analytically in the case of a viscous fluid confined between two stationary, rigid, parallel plates at $z=0$ and $z=h$^24^.

To apply these results to hydrodynamic simulations of scan path multiplexing, we follow the method presented in our recent study that used multiple scan paths to achieve net flows but with reduced time-averaged temperature gradients^39^.

First, we summarize the case of a single scan path. During one scan (i.e., for $-l/U\le t\le l/U$), a heat spot translates from $x=-l$ to $x=l$ along the line $y=0$. Motivated by experimental measurements^20,21,29^, we model the effect of the laser as a translating Gaussian profile for an instantaneous temperature change $\Delta T\left(x,y,z,t\right)$ in the fluid. The instantaneous temperature field $T\left(x,y,z,t\right)$ during one scan may therefore be written as $T\left(x,y,z,t\right)={T}_{0}+\varDelta T\left(x,y,z,t\right)$ with $\varDelta T\left(x,y,t\right)=\varDelta {T}_{0}A\left(t\right)\exp \{-\left[{\left({\rm{x}}-{\rm{Ut}}\right)}^{2}+{{\rm{y}}}^{2}\right]/2{{\rm{a}}}^{2}\}$, where ${T}_{0}$ is the reference temperature, $\Delta {T}_{0}$ is the maximum temperature change, $a$ is the characteristic radius of the heat spot, $U$ is the constant speed of translation of the heat spot, and $A\left(t\right)$ is its dimensionless, time-dependent amplitude.

Due to the localized laser heating, the fluid undergoes thermal expansion and thermal viscosity changes locally. Since the temperature changes are small, so are the local changes in the fluid density $\rho$ and the viscosity $\eta$. They may thus be modelled using standard, linear relationships,

1 $$\rho ={\rho }_{0}\left(1-\alpha \varDelta T\right)$$

2 $$\eta ={\eta }_{0}\left(1-\beta \varDelta T\right)$$

where ${\rho }_{0}$ and ${\eta }_{0}$ are the reference density and viscosity of the fluid at temperature ${T}_{0}$, $\alpha$ is the thermal expansion coefficient and $\beta$ is the thermal viscosity coefficient. The resulting instantaneous, inertialess fluid flow gives rise to the net displacement, after a full laser scan, of tracer particles in the fluid.

For the glycerol-water mixtures used in our experiments, we assume the thermal viscosity coefficient $\beta$ to be much larger than the thermal expansion coefficient $\alpha$. In this case, and in the mathematical limit ${\rm{\alpha }}\Delta {{\rm{T}}}_{0},{\rm{\beta }}\Delta {{\rm{T}}}_{0}\ll 1$, the leading-order net displacement of a tracer particle with initial position ${{\bf{X}}}_{0}\equiv \left({{\rm{X}}}_{0},{{\rm{Y}}}_{0}\right)$ in the fluid, due to one scan of the heat spot, is given analytically by

3 $$\Delta {\bf{X}}\left({{\bf{X}}}_{0}\right)=\alpha \beta \varDelta {T}_{0}^{2}{\int}_{\!-{t}_{0}}^{{t}_{0}}{{\bf{u}}}_{1,1}\left({{\bf{X}}}_{0},t\right){\rm{d}}t$$

where ${t}_{0}\equiv l/U$ is half the scan period and ${{\bf{u}}}_{1,1}$ is the instantaneous fluid velocity field at order $\alpha \beta$^24^. The flow ${{\bf{u}}}_{1,1}$ at order $\alpha \beta$ during one scan is given analytically by

4 $$\begin{array}{l}{{\bf{u}}}_{1,1}\left({\bf{x}},t\right)={A\left(t\right)}^{2}U\left\{{{\bf{e}}}_{{\rm{x}}}\left\{\frac{{a}^{2}}{4{r}^{2}}-\frac{{a}^{2}{\left(x-{Ut}\right)}^{2}}{2{r}^{4}}+\left[-\frac{{a}^{2}}{2{r}^{2}}+\frac{{a}^{2}{\left(x-{Ut}\right)}^{2}}{{r}^{4}}\right]\exp \left(-{r}^{2}/2{a}^{2}\right)\right.\right.\\\qquad\qquad\quad+\left.\left[\frac{{a}^{2}}{4{r}^{2}}-\frac{{a}^{2}{\left(x-{Ut}\right)}^{2}}{2{r}^{4}}\right]\exp \left(-{r}^{2}/{a}^{2}\right)-\frac{1}{4}{\text{E}}_{1}\left({r}^{2}/2{a}^{2}\right)+\frac{1}{4}{\text{E}}_{1}\left({r}^{2}/{a}^{2}\right)\right\}\\\qquad\qquad\quad+\left.{{\bf{e}}}_{{\rm{y}}}\left[-\frac{{a}^{2}(x-{Ut})y}{2{r}^{4}}+\frac{{a}^{2}(x-{Ut})y}{{r}^{4}}\exp\left(-{r}^{2}/{2a}^{2}\right)-\frac{{a}^{2}(x-{Ut})y}{2{r}^{4}}\exp \left(-{r}^{2}/{a}^{2}\right)\right]\right\}\end{array}$$

where $r\equiv {\left[{\left(x-{Ut}\right)}^{2}+{y}^{2}\right]}^{1/2}$ is the distance to the center of the heat spot at time $t$ and ${\text{E}}_{1}$ is the exponential integral, given by

5 $${\text{E}}_{1}\left(z\right)\equiv {\int}_{\!z}^{\infty }\frac{\exp \left(-s\right)}{s}{\rm{d}}s$$

As in previous work^39^, we may then obtain the net displacement of a tracer particle due to a scan path with arbitrary position and orientation by translating and rotating the result for the individual scan path obtained above. To leading order (i.e., assuming again $\alpha \Delta {T}_{0},\beta \Delta {T}_{0}\ll 1$), the net displacement of a tracer due to sequential translation of the laser during multiplexing (i.e., multiple scan paths) is obtained as the sum of the net displacements due to each of the scan paths; the average velocity field of tracer particles is given by the total net displacement divided by the scan period. The streamlines of the average velocity of tracers provide the trajectories of the tracer particles due to repeated scanning of the laser (Fig. 3e, f and Supplementary Videos 8 and 9), in the same limit. In Fig. 3e, the dimensionless speeds indicated by the color bar are scaled by $\alpha \beta \Delta {T}_{0}^{2}U$; thus, they hold for arbitrary values of these parameters.

In our simulations, based on experimental observations, we use a characteristic heat-spot radius $a=4 \, {\rm{\mu }}{\rm{m}}$ in good agreement with experiments^29^ and we pick the heat-spot amplitude to be sinusoidal, given by

6 $$A\left(t\right)={\cos }^{2}\left(\frac{\pi t}{2{t}_{0}}\right)$$

for $$-{t}_{0}\le t\le {t}_{0}$$^24^.

### Algorithm for context-aware local decisions to ‘push’ or ‘pull’

As described above, in the feedback loop the position of the laser scan path is usually determined such that the path is parallel to the connecting line of particle and associated target and positioned behind the target so that the generated thermoviscous flows ‘push’ the particle towards the target. To reduce cross-talks between the alignment of adjacent particles, we devised an algorithm that makes a context-aware decision whether to use pushing or pulling flows: The algorithm decides between pushing and pulling flows based on the distance of the scan path center to the surrounding particles. If the distance to the closest particle (that is not the intended target of the manipulation) is lower for pushing than for pulling flows, the algorithm will apply pulling flows and vice versa. If the distances are equal, pushing flows will be applied. This algorithm will reduce large unintended displacements of particles since the flow speed decays with the distance to the center of the scan path^22^.

See also Supplementary Video 10 for a visualization.

### Design of motion patterns

The motion pattern of the humanoid robot (Fig. 3f and Supplementary Video 5) was inspired by a video about the work of Johansson^40,41^. Motion patterns of robots displaying emotions (Fig. 4e and Supplementary Videos 8 and 9) were modelled after the work of Troje^28,42,43^.

### Fluorescence-based temperature measurements

To characterize the temperature change in the sample induced by the scanning of the infrared laser (Fig. S4), we built chambers containing Rhodamine B dye as shown before by Mittasch et al. ^29^. The quantum yield, and hence the emission rate, of Rhodamine B reduces with increase in temperature and can therefore be used to characterize temperature changes in a system.

The Rhodamine B molecules were immobilized in a UV curable polyacrylamide gel to prevent dye accumulation/depletion due to thermophoresis. To prepare the UV curable gel, we used DMPA (2,2-Dimethoxy-2-phenylacetophenone, CAS number: 24650-42-8, Sigma Aldrich) as UV-photoinitiator for the gel polymerization. Specifically, 30 mg of DMPA were gently dissolved in 0.5 mL of ethanol and then mixed with 3.75 mL of 40% (m/v) AAm (Acrylamide Solution, catalog #: 1610140, Bio-rad), 3.75 mL of 2% (m/v) MBAAm (Bis Solution, catalog #: 1610142, Bio-rad) and 2 mL of distilled water. 3.3 mg of Rhodamine B molecules (Acryloxyethyl thiocarbamoyl Rhodamine B, CAS number: 1821380-54-4, Polysciences) were previously dissolved in 8.25 mL of 40% (m/v) AAm. The final concentration of DMPA, Aam, Rhodamine B molecules and MBAAm are 0.3% (m/v), 15% (m/v), 0.014% (m/v) and 0.75% (m/v), respectively.

0.1 μL of 5 μm diameter fluorescent beads based on melamine resin (75908-10ML-F, Sigma-Aldrich) was added to 9.9 μL of Rhodamine B UV curable gel and 2 µL of this solution were spread onto an objective slide (sapphire or conventional glass), sandwiched with a cover glass and sealed with nail polish to form a chamber as described before. The chamber was exposed for 1 min to 365 nm wavelength light (LED 360 mW, Thorlabs M362L2) to polymerize the polyacrylamide gel. Then it was sealed with nail polish.

To determine the relation between ambient temperature and relative intensity of the dye (Fig. S4a), the ambient temperature in the chamber was controlled using a custom-build thermal stage. This stage consists of a highly thermally conductive sapphire slide (model SMS-7521, UQG Optics) that replaces the objective slide in our chamber construction and is actively cooled by two proportional-integral-derivative controlled Peltier elements (model TES1-127021, TEC, Conrad).

To minimize the effect of photobleaching of Rhodamine B, measurements of the dye intensity used to characterize the temperature change due to the IR light (Fig. S4) were averaged over acquisition periods of 10.2 s before and after the IR laser was switched on. We measured that the intensity reduction due to photobleaching in the same time period only amounts to 0.06 °C, which is negligible compared to the measured heating of several °C (Fig. S4d, e). During the measurements of intensity at different ambient temperatures (Fig. S4a) the excitation light was switched off for ca. 2 min between acquisitions to allow recovery of fluorescence.

### Use of scientific software

We used ImageJ Fiji^44^ to process microscopy images and Fiji’s plugin TrackMate^45^ to visualize and analyze particle trajectories (Figs. S1, S3 and Supplementary Video 3).

### Supplementary information

Supplementary Information

Supplementary Video 1

Supplementary Video 2

Supplementary Video 3

Supplementary Video 4

Supplementary Video 5

Supplementary Video 6

Supplementary Video 7

Supplementary Video 8

Supplementary Video 9

Supplementary Video 10

## Data Availability

The data underlying the results presented in this paper is available from the corresponding author upon reasonable request.
