# Supplementary material for: Opto-fluidically multiplexed assembly and micro-robotics
Source: Light Sci Appl. 2024 Feb 27;13:59. doi: 10.1038/s41377-024-01406-4 (PMC10897173; doi:10.1038/s41377-024-01406-4)
Supplement: Supplementary file 1 — Supplementary Information [file 41377_2024_1406_MOESM1_ESM.docx]

**Supplementary Information for**

**“Opto-fluidically multiplexed assembly and micro-robotics”**

*Elena Erben^1,2,3^, Weida Liao^4^, Antonio Minopoli^1,2^, Nicola Maghelli^1,2^,*

*Eric Lauga^4^, Moritz Kreysing ^1,2,3^**

1) Max Planck Institute of Molecular Cell Biology and Genetics, Dresden, 01307, Germany

2) Centre for Systems Biology, Dresden, 01307, Germany

3) Institute of Biological and Chemical Systems – Biological Information Processing. Karlsruhe Institute of Technology (KIT), Eggenstein-Leopoldshafen, 76344, Germany

4) Department of Applied Mathematics and Theoretical Physics, University of Cambridge,

Cambridge, CB3 0WA, UK

**) Correspondence: moritz.kreysing@kit.edu*

**Contents**

| Fig. S1: Deviation of a single particle from a star-shaped trajectory. | 3 |
| --- | --- |
| Fig. S2: Measure of constant power deposition per scan path as used in Fig. 3a-c. | 4 |
| Fig. S3: Velocity along individual scan paths increases with scan path multiplexing. | 5 |
| Fig. S4: Induced heating per laser scan path increases with path-multiplexing in a classic glass-walled chamber, but remains constant in a sapphire-walled chamber. | 6 |
| Fig. S5: Manipulation of particles is possible on particles of sizes between 0.2 and 19.3 µm. | 8 |
| Fig. S6: Manipulation of particles is possible independent of medium viscosity. | 9 |
| Fig. S7: Manipulation of particles of different material properties. | 10 |
| Fig. S8: Detailed description of the feedback loop used for positioning multiple particles. | 11 |
| Supplementary Table 1: Comparison of our opto-fluidic approach to other existing methods for colloid positioning. | 12 |
| Supplementary Video 1: Iterative alignment of 2 particles to a distance of 5 µm as shown in Fig. 2a. | 13 |
| Supplementary Video 2: Assembly of a triangular pattern from 3 particles demonstrating the mechanism of iterative alignment of multiple particles in the order of distance to target. | 14 |
| Supplementary Video 3: Dynamic positioning of a particle along a star-shaped path. | 15 |
| Supplementary Video 4: Mechanism of scan path multiplexing for the hexagonal pattern from Fig. 3a-c. | 16 |
| Supplementary Video 5: Assembly of 13 randomly distributed particles into the shape of a humanoid figure that then is made to perform jumping jacks. | 17 |
| Supplementary Video 6: 2 particles approaching each other and being separated through pushing and pulling flows as in Fig. 4a and b. | 18 |
| Supplementary Video 7: Videos showing difficulty of using only pushing flows to manipulate multiple particles. | 19 |
| Supplementary Video 8: Particles being controlled by 8 scan paths to form patterns of walking humanoid figures that display different stereotypical character traits (female, male, sad, nervous). | 20 |
| Supplementary Video 9: Rapidly changing flow fields enabling the manipulation of the ‘female’ humanoid robot. | 21 |
| Supplementary Video 10: Algorithm for context-aware local decisions to ‘push’ or ‘pull’. | 22 |
| References | 23 |


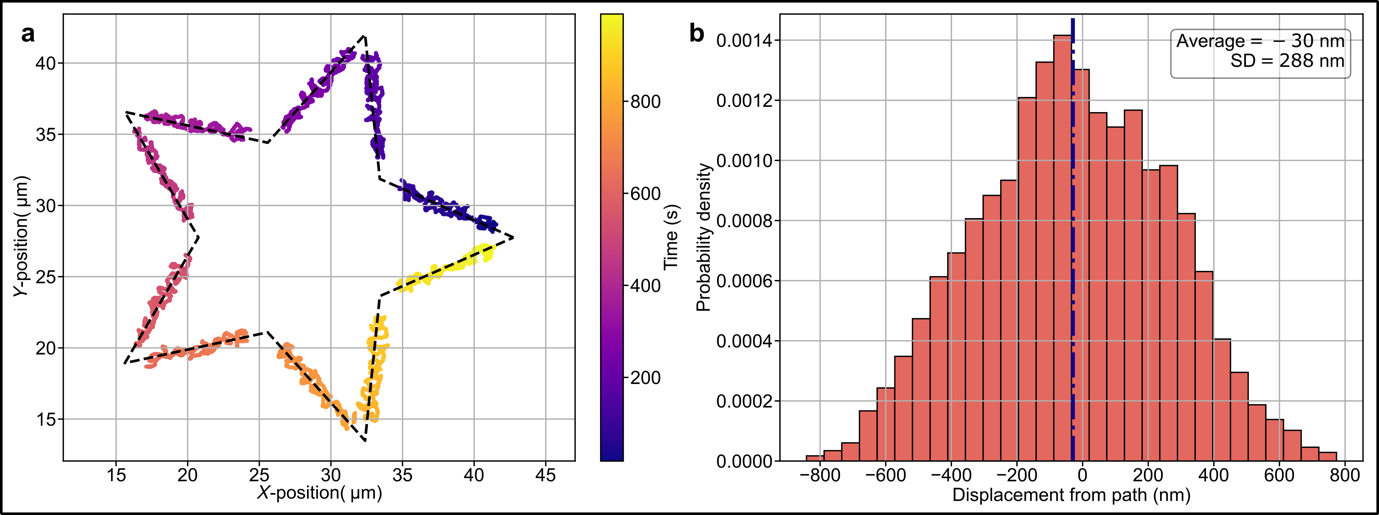


**Fig. S1: Deviation of a single particle from a star-shaped trajectory.**

Fig. 2d shows a single particle being guided along a star-shaped path via translation of the target. (**a**) For each particle position, we measured the displacement from the path. Particle positions within 1.5 µm of the star’s corners were excluded to avoid skewing of the data to lower distance values. (**b**) The histogram shows the probability density of the displacement from the star-shaped path revealing a standard deviation (SD) of 288 nm. The average displacement is marked with the blue dashed line. The particle’s track was derived using Fiji’s tracking plugin TrackMate^1^.

**
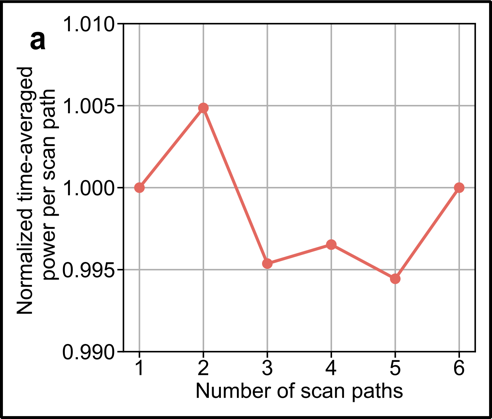
**

**Fig. S2: Measure of constant power deposition per scan path as used in Fig. 3a-c.**

Measurements of the laser power (**a**) during the scan path multiplexing measurements as described in Figs. 3a-c confirm that the laser powers chosen for the experiments deviate less than 1% from a linear increase with the number of scan paths.

**
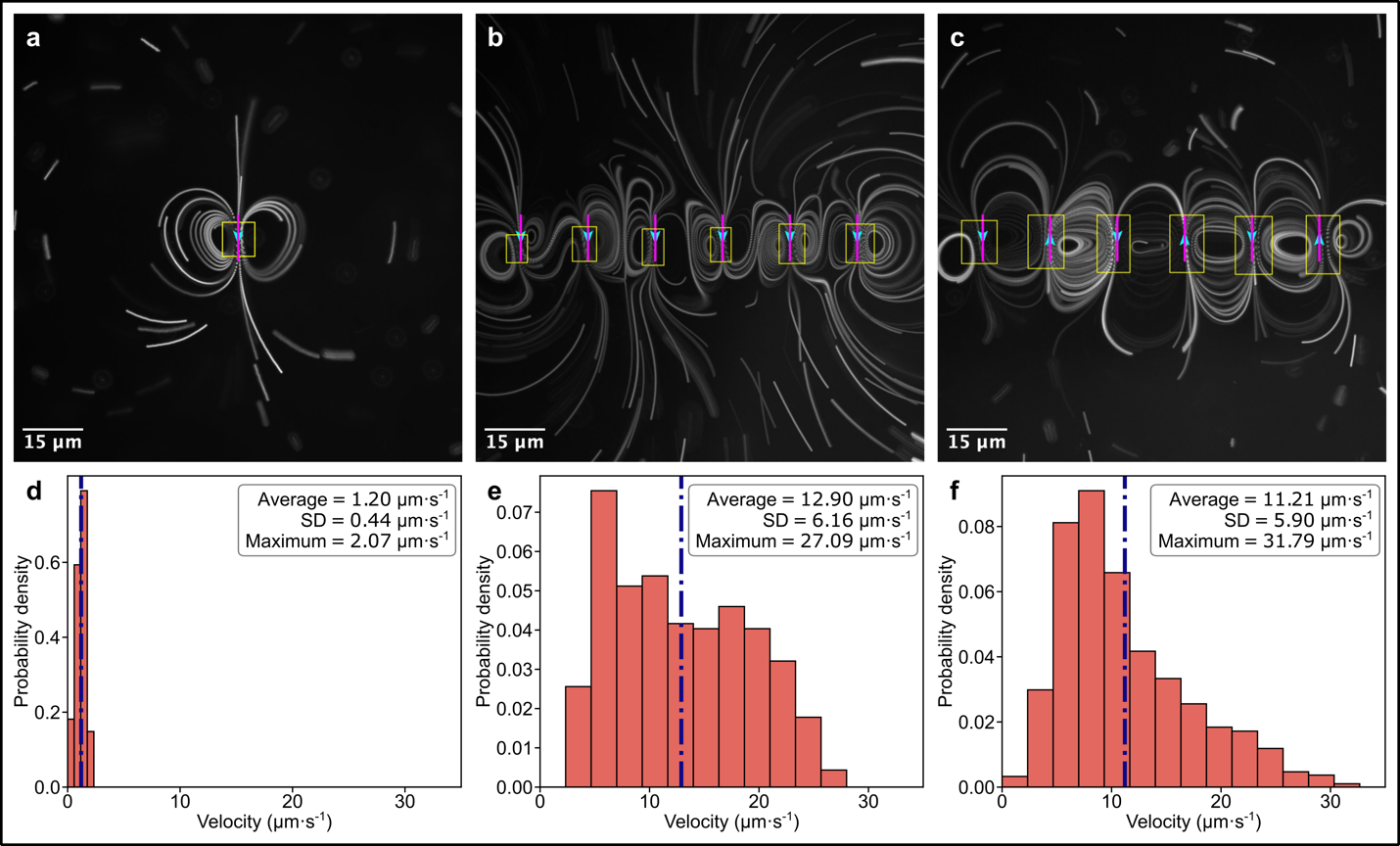
**

**Fig. S3: Velocity along individual scan paths increases with scan path multiplexing.**

Speed of 0.5 µm diameter fluorescent particles in pure honey (25 µm chamber thickness) when stimulated by a single scan path (**a** and **d**), six scan paths pointing in the same direction (**b** and **e**), and six scan paths pointing in alternating directions (**c** and **f**). Between single and six scan paths the scan frequency was kept constant, but the laser power was increased 6-fold to keep the energy released per line constant. In order to avoid distortion of the flow fields we symmetrized the sequence of line scans, we scanned the lines in the following order: 1, 4, 2, 5, 3, 6; which marks one period of the scan pattern. **a**, **b** and **c** show the particle trajectories in a time projection with the laser scan path indicated in pink and the flow direction in blue. The particle velocities were tracked in proximity to the scan path (area marked by yellow rectangles) using Fiji’s tracking plugin TrackMate^1^. Histograms of the speed are shown in **d**, **e** and **f**, respectively. The histograms show a clear increase in the average speed (indicated by a blue dashed line) of approximately 10 moving from single (**d**) to six scan paths (**e**). The average speed does not differ greatly between six co-directional (**e**) and six alternatingly directed scan paths (**f**). This suggests that the speed-up along a path does not originate from the flows generated on neighboring scan paths.

In **d**, **e** and **f**, SD is the standard deviation.

**
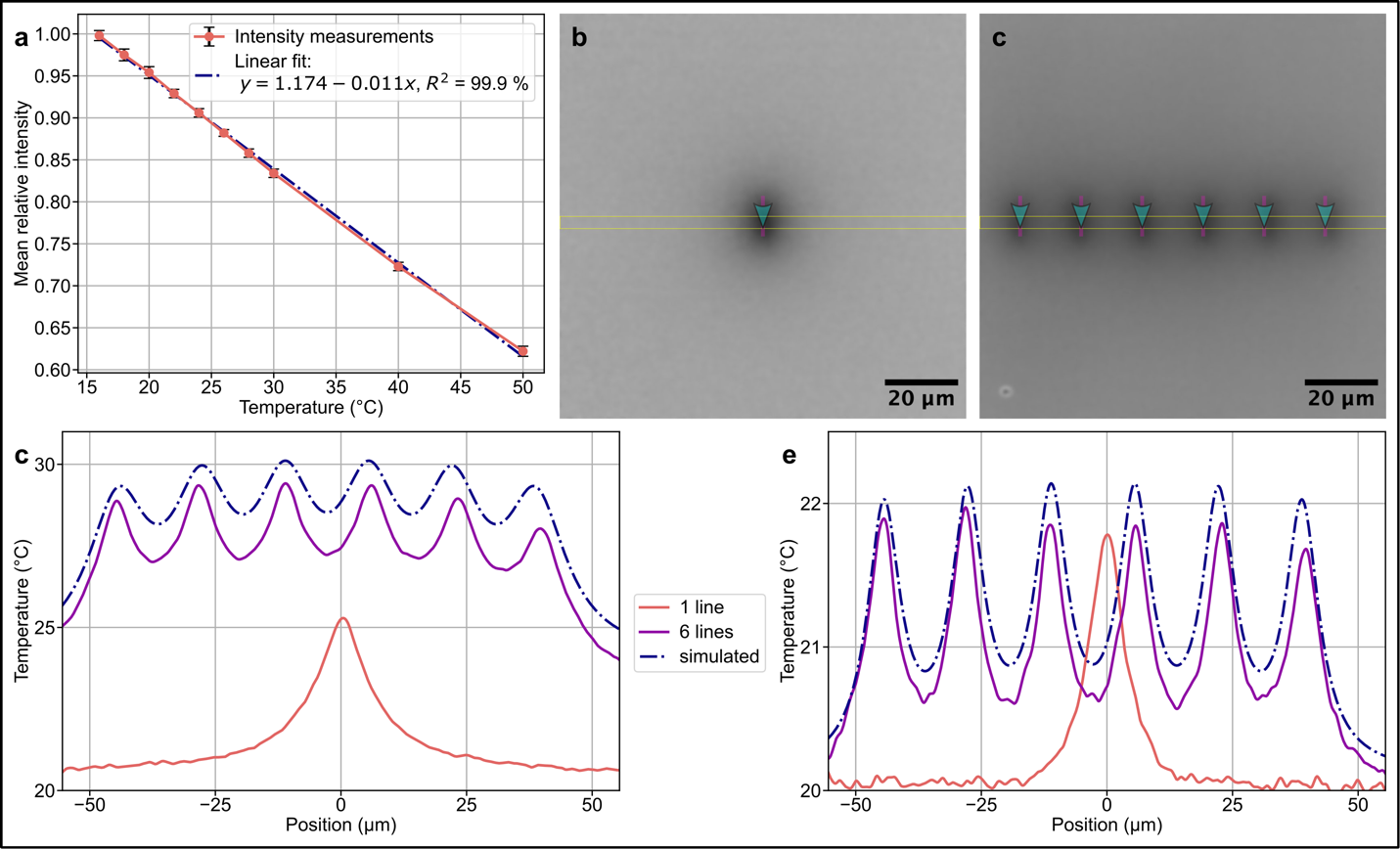
**

**Fig. S4: Induced heating per laser scan path increases with path-multiplexing in a classic glass-walled chamber, but remains constant in a sapphire-walled chamber.**

**a**, In the range of 15 to 50 °C the mean relative emission intensity of the Rhodamine B dye changes linearly with temperature (-0.011 K^-1^). The intensity is averaged over the area of interest, the error bars show the standard deviation. Normalized time-averaged images of Rhodamine B samples when exposed to an IR laser spot scanning along **b**, a single line and **c**, along six lines, respectively. The six lines are scanned at the same frequency as a single line with 6-fold laser power in a glass-walled chamber. A Gaussian blur filter with 0.56 µm diameter was applied to filter out any non-temperature related noise. **d**, Intensity cross sections in *X*-direction (perpendicular to the scan lines) and averaged over 3.33 µm (yellow boxes in **a** and **b**). The overall temperature is increased for six lines compared to the single one due to the relatively slow heat dissipation in the glass-walled chamber and to the subsequent superimposition of the long tails of the temperature profile. We rebuilt the temperature profile arising from the superimposition of individual heat stimuli (whose profile was delineated by scanning along a single line as shown in **b** being arranged along six lines as shown in **c**. Specifically, we fitted the temperature profile measured for the single line with a Cauchy-Lorentz equation and we superimposed the curves with the peak shifted to different locations corresponding to those of the experiment with six lines as in **b**. The result (dashed green line) closely resembles the measurement for six lines. This confirms that the heating induced by multiple scan paths is indeed a superimposition of the heating induced by six single scan paths. However, due to the temperature-increase in the entire chamber induced by a single line, the overall temperature is much elevated for six scan paths (peak increase of 10 °C). **e**, We repeated the experiment in a sapphire-walled chamber with superior heat dissipation properties. In this setting the peak temperature increase can be reduced to 2 °C for a single scan path without heating of the entire chamber. In addition, the much higher dissipation coefficient of sapphire avoids the formation of the long tails in the temperature profile. For six scan paths the peak temperature remains the same as for a single path. However, the temperature is still increased between the scan paths due to the superimposition of the heat profiles. The exact heat distribution in the sample will depend on the distribution of the scan paths.

**
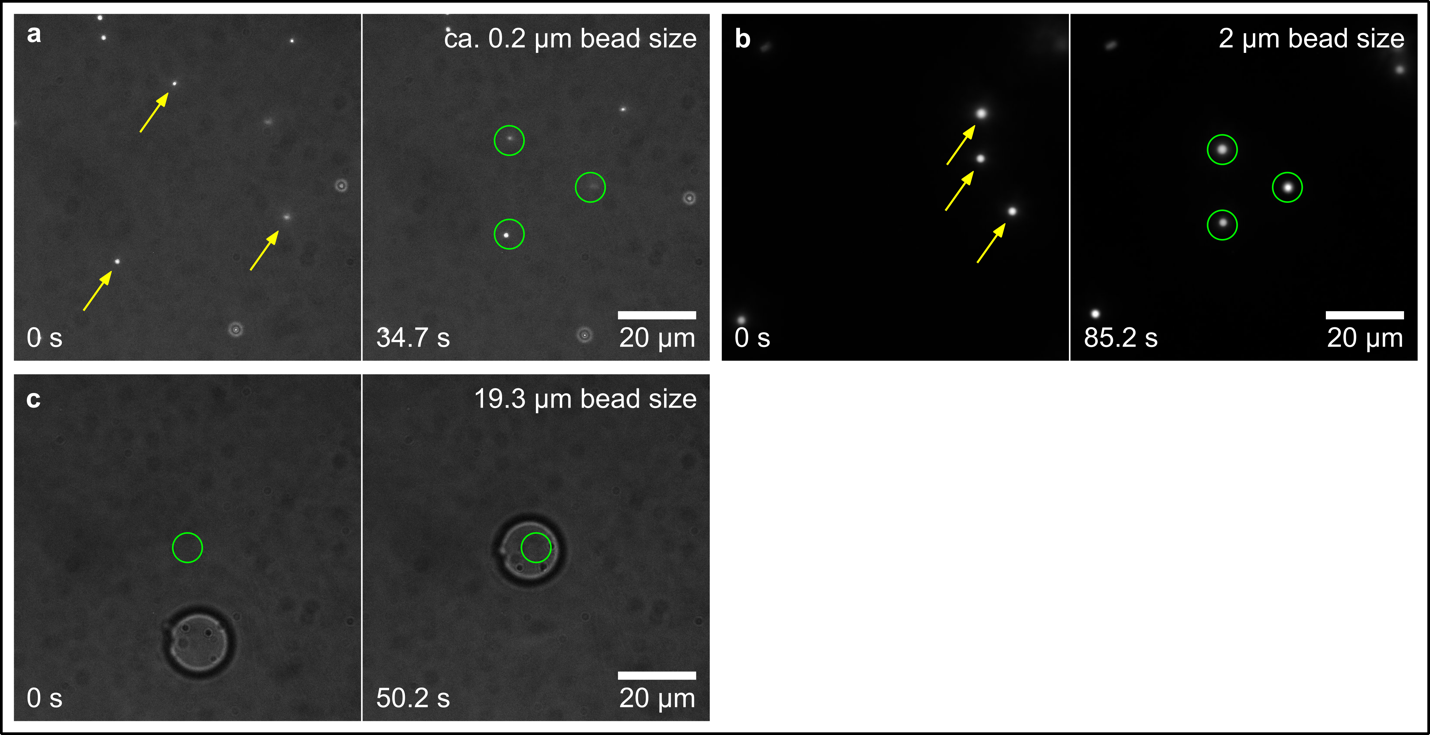
**

**Fig. S5: Manipulation of particles is possible on particles of sizes between 0.2 and 19.3 µm.**

Positioning of microparticles of sizes **a,** 0.2 **b,** 2 and **c,** 19.3 µm in glycerol in the form of equally sided triangles **a**,**b** or reach a predefined destination **c**. Yellow arrows mark the selected particles in the image before positioning where necessary.

**
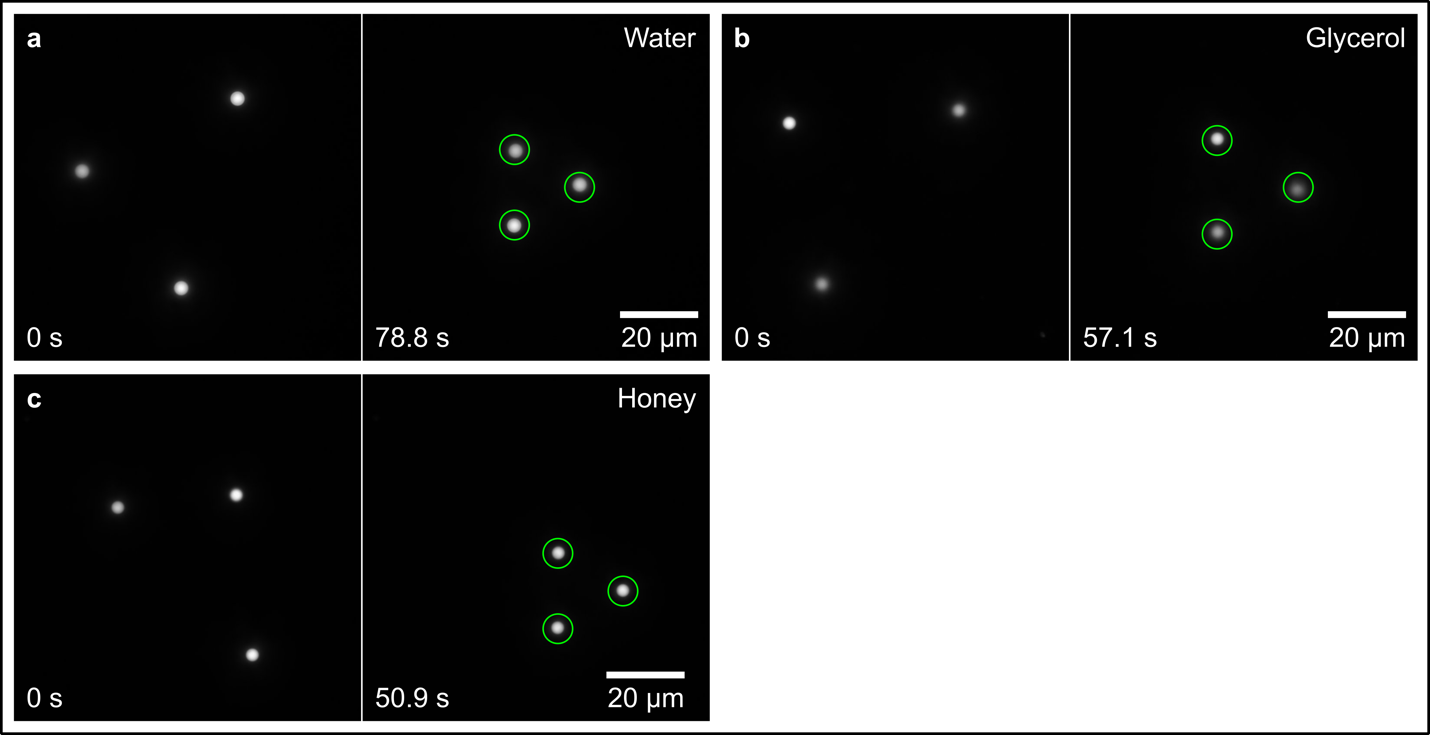
**

**Fig. S6: Manipulation of particles is possible independent of medium viscosity.**

Positioning of PS microparticles (3 µm diameter) is shown in **a,** water, **b,** glycerol and **c,** honey.

**
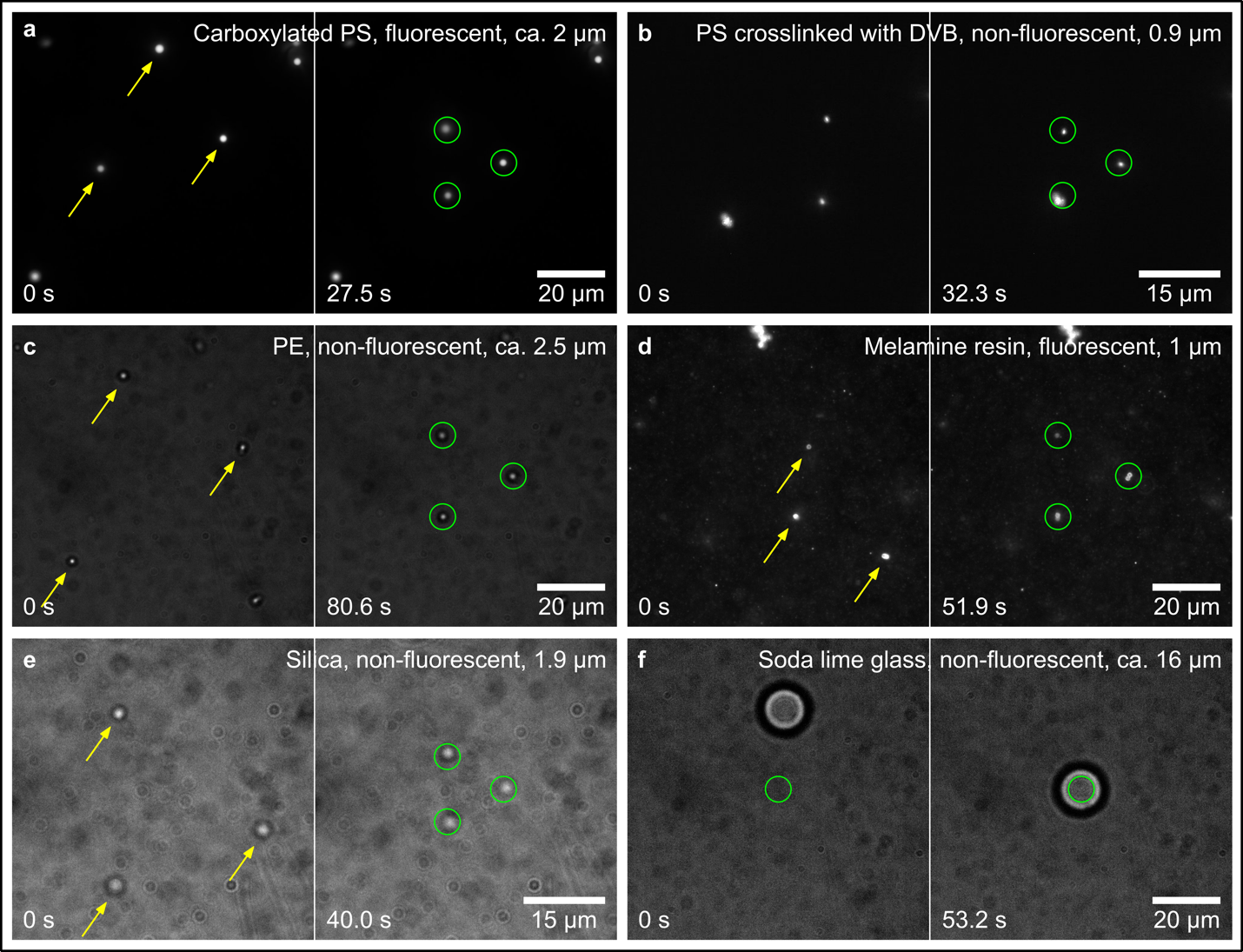
**

**Fig. S7: Manipulation of particles of different material properties.**

We show successful automatic positioning of particles is possible for different particle materials and with surface modifications: **a,** Carboxylated PS, **b,** PS crosslinked with divinylbenzene (DVB), **c,** polyethylene (PE), **d,** particles based on Melamine resin (including one duplet of beads), **e)**, silica and **f,** soda lime glass. These experiments also show manipulations are possible on beads of different sizes (adding to our results shown in Extended Data Fig. 6) and the detection algorithm also works on non-fluorescing samples. Yellow arrows mark the selected particles in the image before positioning where necessary.


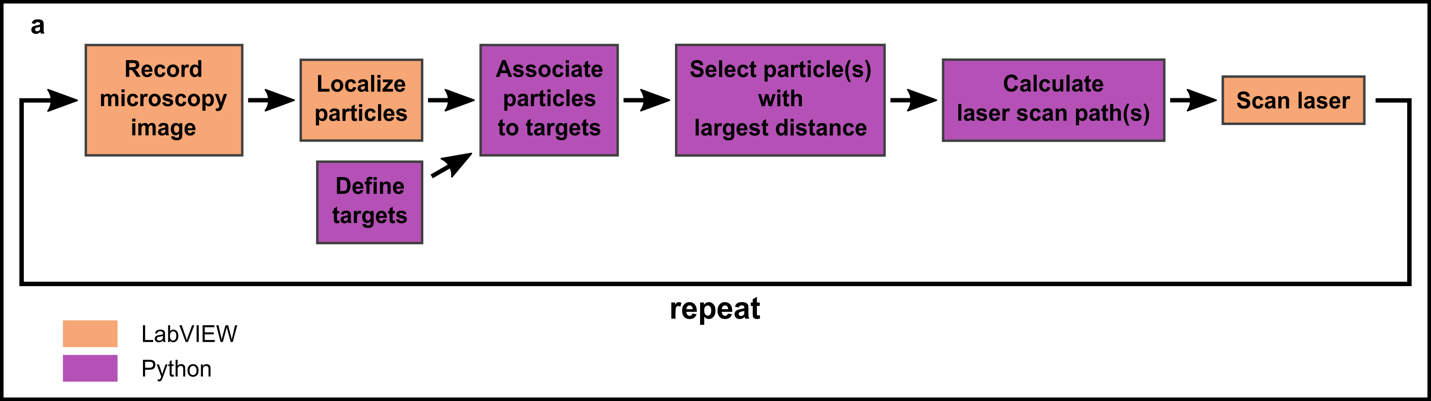


**Fig. S8: Detailed description of the feedback loop used for positioning multiple particles.**

**a**, The loop consists of 6 steps, some of which are carried out in LabVIEW and the rest in Python. For a detailed description of the steps, see methods section.

| Method and literature examples | Mechanism | **Optical control** | Typical Power density / field strengths | **Laser exposure free** | Multiplexed trapping / manipulation possible? | Common particle size range | Typical Spatial resolution | Dimensional control (typical) | Material requirements for particles | Typical positioning  velocity |
| --- | --- | --- | --- | --- | --- | --- | --- | --- | --- | --- |
| Feedback-controlled thermoviscous flows (^2,3^ and this work) | Feedback-controlled flows induced by rapid IR laser scanning | Yes | 2⋅10^5^ W⋅cm^-2^ at beam focus, but indirect manipulation possible | Yes | Yes (shown for up to 15 particles) | 0.2 – 20 µm | 25 nm | 2D | No requirements | 1 µm⋅s^-1^ to 10 µm⋅s^-1^ and quadratically scaling with ΔT |
| Optical Tweezers^4–8^ | Optical gradient produced by a highly focused beam | Yes | 10^5^ – 10^8^ W⋅ cm^-2^ | No | Yes ( > 100 objects using acousto-optic deflectors or galvanometer-driven mirrors) | 10 nm – 1 mm | 0.1 – 1 nm | 3D | High refractive index contrast between particle and surrounding liquid | 0.1 mm**⋅**s^-1^ |
| Magnetic Tweezers^7,9,10^ | Electromagnetic field gradients produced by permanent magnets or electromagnets | No | 1 – 10 T | Yes | Strongly limited due to challenges to dynamically localize magnetic fields at a distance | 100 nm – 10 μm | 1 – 10 nm | 3D | Magnetic particle | 0.1 mm**⋅**s^-1^ |
| Electrokinetic Tweezers^11–15^ | Feedback-controlled voltage applied to the solution to produce electrophoretic drift | No | 10^4^ – 10^7^ V⋅m^-1^ | Yes | Yes (up to 2 particles) | 1 nm – 1 mm | 0.1 – 1 μm | 3D | No | 3 mm⋅s^-1^ |
| Acoustic Tweezers^16–20^ | Acoustic radiation force based on standing-wave, traveling-wave or acoustically induced flows | No | 10^-2^ – 10 W⋅ cm^-2^ | Yes | Yes (up to 12 particles) | 100 nm – 10 mm (with resolution limited by the wavelength of sound) | 1 – 10 μm | 3D | No strong requirements, potentially density and compressibility | 1 mm⋅s^-1^ |
| Hydrodynamic Tweezers^21–24^ | Laminar flows produced by pressure-driven microfluidic systems | No | n.a. | Yes | Yes (up to 6 particles) | 100 nm – 1 mm | 1 – 10 μm | 3D | No requirements | 100 µm⋅s^-1^ |
| Thermophoresis^25–27^ | Thermal gradient | No | 10^3^ – 10^4^ W⋅ cm^-2^ | Yes | Yes | 1 – 10 μm | 100 nm | 2D | No requirements | 1 µm⋅s^-1^ |
| Optoelectronic Tweezers^28–31^ | Optical tweezers combined with electrode-based dielectrophoresis | Yes | 10^-2^ – 10 W⋅ cm^-2^ | No | Yes | 100 nm – 10 μm | 1 – 10 μm | 3D | Dielectrophoretic contrasts | 100 µm⋅s^-1^ |
| Plasmonic Tweezers^32–35^ | Plasmonic field trapping produced by surface plasmon polaritons and localized surface plasmons | Yes | 10^2^ – 10^4^ W⋅ cm^-2^ | No | Yes (up to 15 particles) | 10 nm – 1 μm | 10 – 100 nm | 2D | Polarizability of particles, commonly given | 0.1 mm⋅s^-1^ |

**S**upplementary Table 1: Comparison of our opto-fluidic approach to other existing methods for colloid positioning.


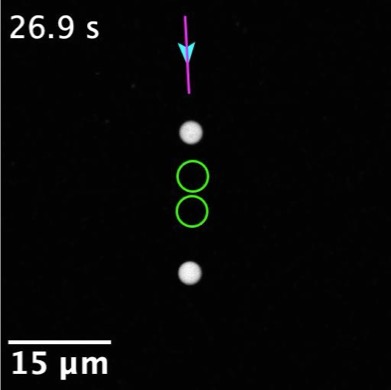


Supplementary Video 1: Iterative alignment of 2 particles to a distance of 5 µm as shown in Fig. 2a.

Targets are shown in green, laser scan path in magenta with direction of flows indicated by blue arrow. The particles are made of fluorescent polystyrene with 3 µm diameter. Despite switching between particles occurring on time scales at which diffusion introduces stochastic components, this approach is still successful.

**
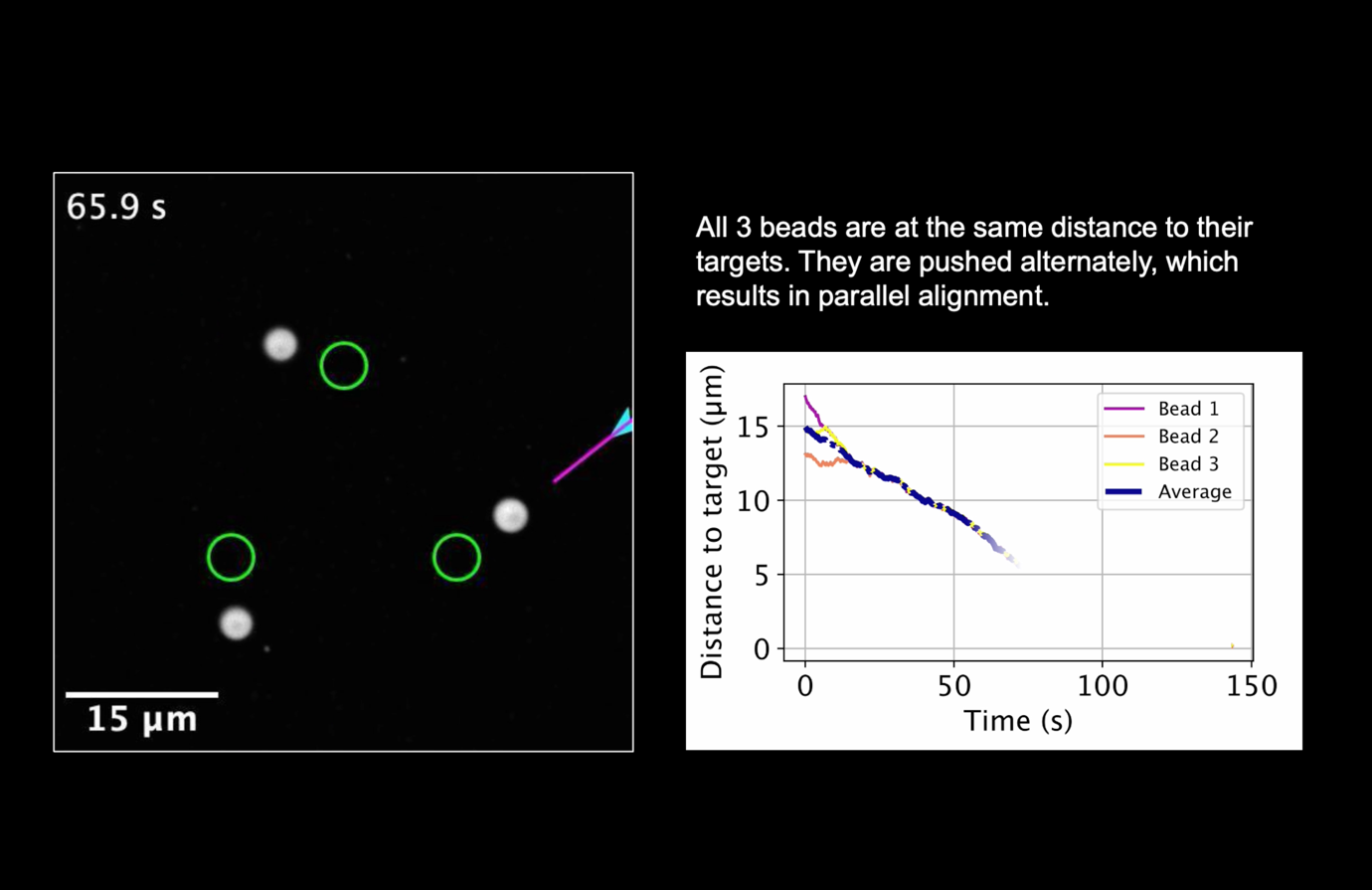
**

Supplementary Video 2: Assembly of a triangular pattern from 3 particles demonstrating the mechanism of iterative alignment of multiple particles in the order of distance to target.

The algorithm is set to position the laser scan path in line with the particle that is currently the furthest from its target, pushing it closer to its target. The situation is reevaluated with every frame (every 50 ms). As shown in the plot, each particle’s distance-to-target collapses onto the average, which decreases linearly with time. Targets are shown in green, laser scan path in magenta with direction of flows indicated by blue arrow. The particles are made of fluorescent polystyrene with 3 µm diameter.


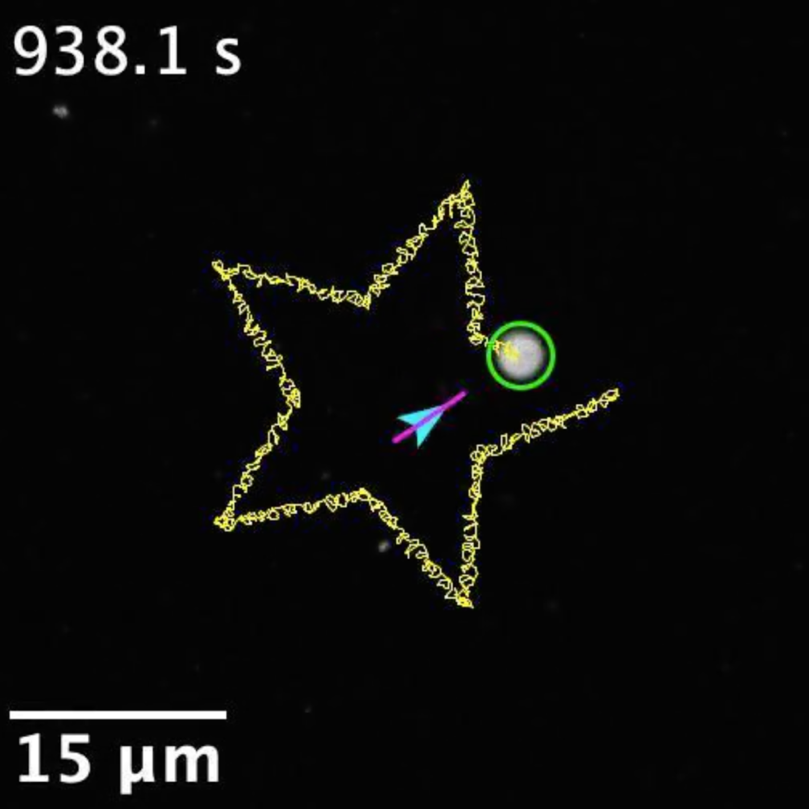


# Supplementary Video 3: Dynamic positioning of a particle along a star-shaped path.

Dynamic positioning is achieved through translation of the target position along a star-shaped path. The particle closely follows the set path (see Fig. S2). Time projection (cropped) shown in Fig. 2d. Target is shown in green, laser scan path in magenta with direction of flows indicated by blue arrow. Particle track created with Fiji’s TrackMate plugin^1^. The particle is made of fluorescent polystyrene with 3 µm diameter.


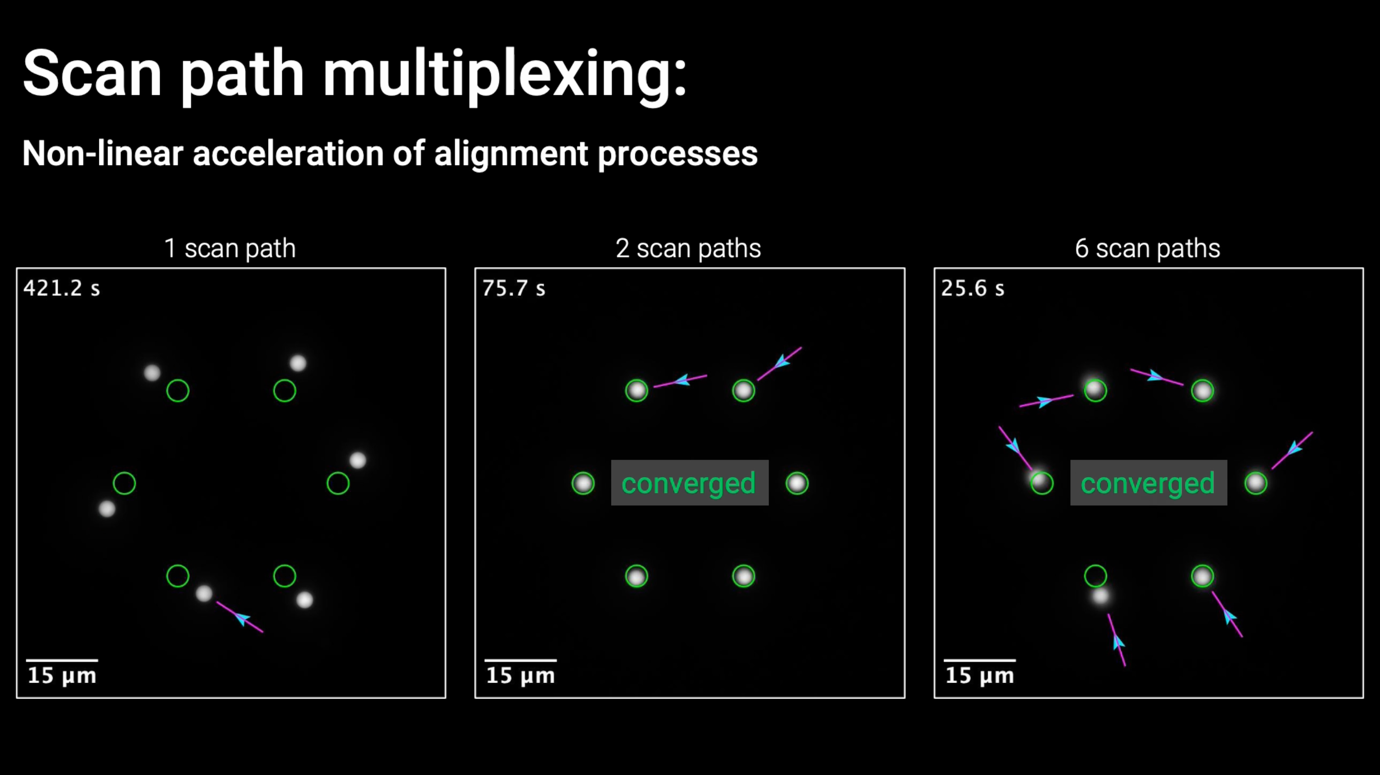


# Supplementary Video 4: Mechanism of scan path multiplexing for the hexagonal pattern from Fig. 3a-c.

Schematic showing the scanning of the laser spot for scan path multiplexing. The laser scan period stays constant leading to an increased scanning speed of the laser spot with more scan paths. The laser power is increased proportionally with the number of scan paths to keep the heat that is deposited at each position constant (see Fig. S5 for temperature measurements). Part 2 of the video shows examples of the data sets used for Fig. 3b and c showing the strong acceleration of the assembly of the hexagon with increased degree of multiplexing.

Targets are shown in green, laser spot in magenta and laser scan paths in magenta with direction of flows indicated by blue arrows. Shown particle and target positions are the same as in Fig. 3a. The particles are made of fluorescent polystyrene with 3 µm diameter.


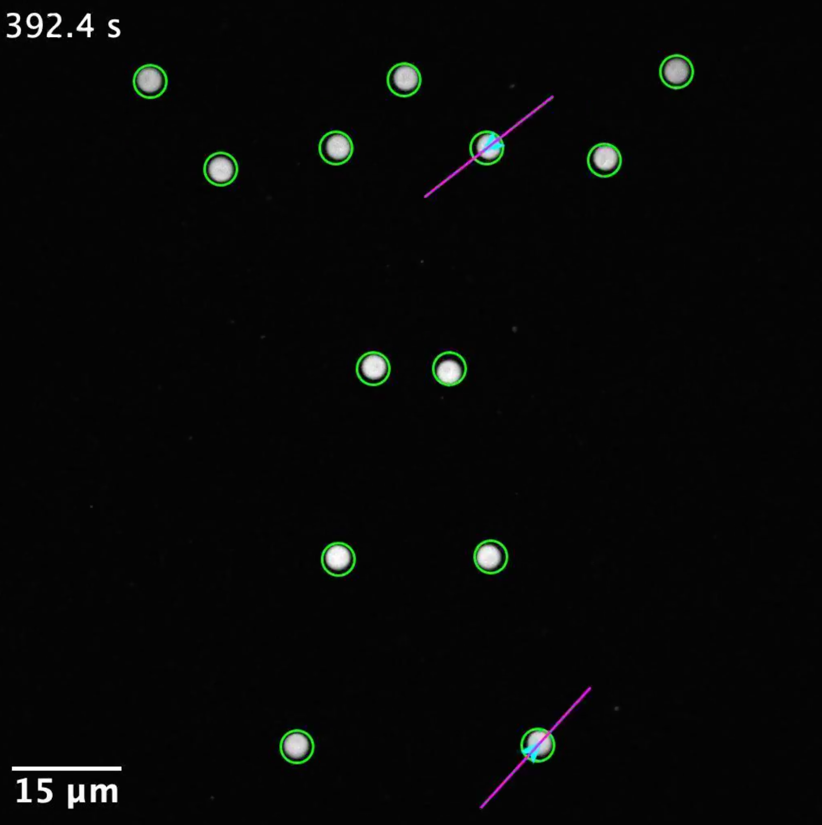


# Supplementary Video 5: Assembly of 13 randomly distributed particles into the shape of a humanoid figure that then is made to perform *jumping jacks*.

The whole manipulation is performed using 2 scan paths. Targets are shown in green, laser scan paths in magenta with direction of flows indicated by blue arrows. The particles are made of fluorescent polystyrene with 3 µm diameter. Images 1 and 2 in Fig. 3f are taken from this dataset.


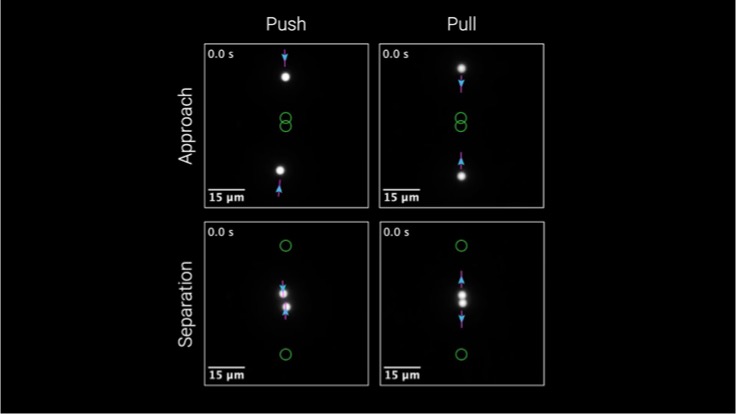


# Supplementary Video 6: 2 particles approaching each other and being separated through pushing and pulling flows as in Fig. 4a and b.

*Pushing* and *pulling* flows require similar amount of time to approach and separate particles, respectively. In contrast to this the time needed to approach the particles using *pulling* flows and separate them using *pushing* flows is greatly increased. This motivates the need for an algorithm that employs context-aware decision (*push or pull*). A systematic analysis of alignment speed is shown in Fig. 4b.

Targets are shown in green, laser scan paths in magenta with direction of flows indicated by blue arrows. The particles are made of fluorescent polystyrene with 3 µm diameter.


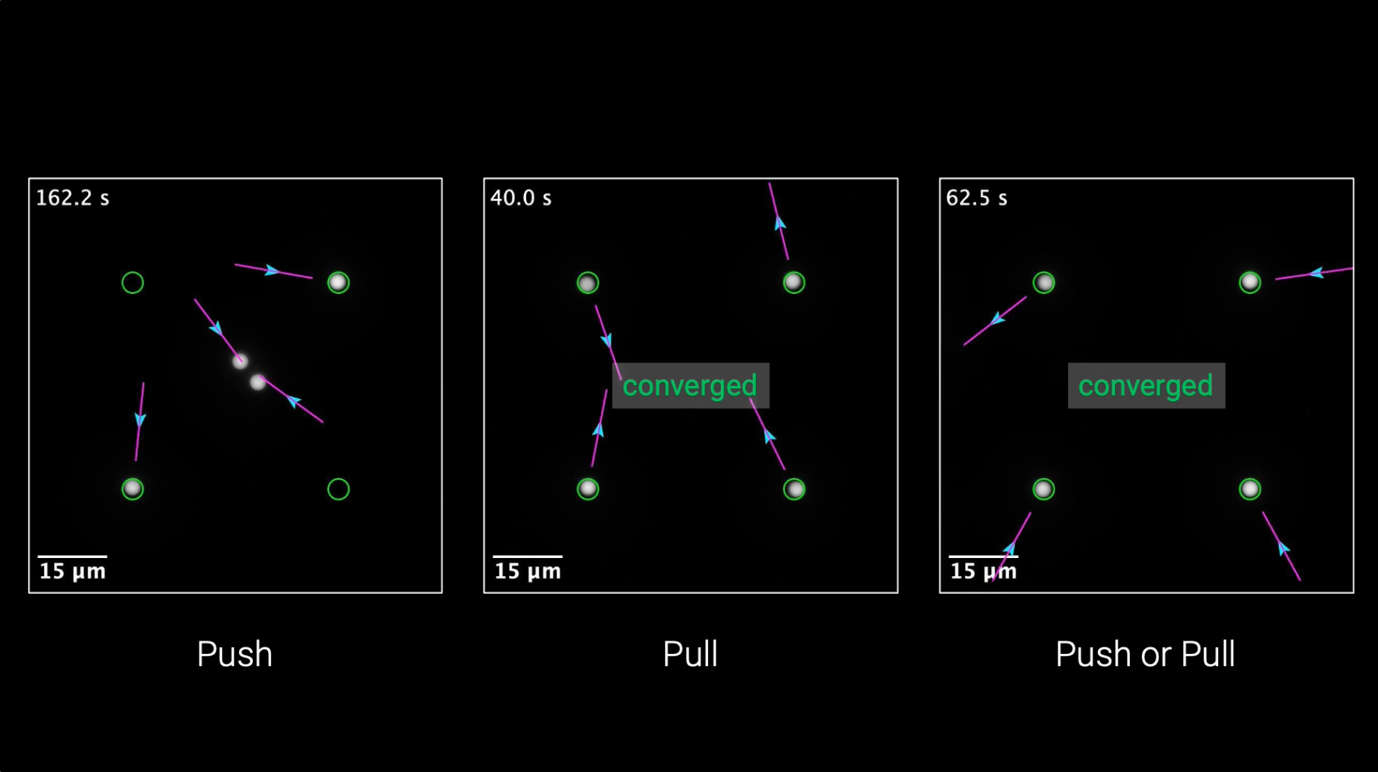


# Supplementary Video 7: Videos showing difficulty of using only pushing flows to manipulate multiple particles.

In the case shown here, the pushing flows needed to assemble a quadratic pattern oppose each other, which effectively locks 2 particles in place and prevents successful assembly. This behavior could neither be observed with only pulling flows nor with the algorithm employing context-aware decision (push or pull), which both achieve assembly of the pattern.

The distance-plots shown in Fig. 4c are derived from these datasets.

Targets are shown in green, laser scan paths in magenta with flow direction indicated by blue arrows. The particles are made of fluorescent polystyrene with 3 µm diameter.


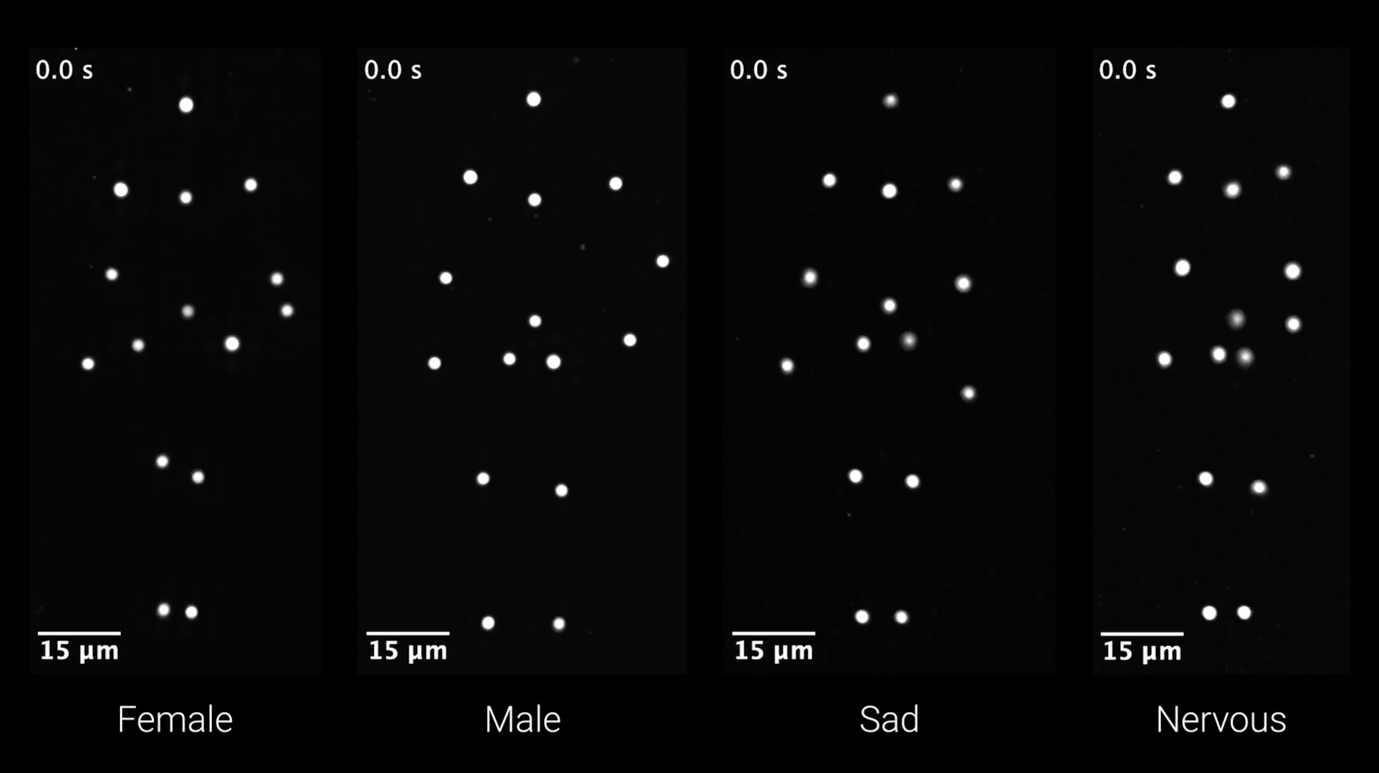


# Supplementary Video 8: Particles being controlled by 8 scan paths to form patterns of walking humanoid figures that display different stereotypical character traits (female, male, sad, nervous).

The first part of the videos shows only the particle movements, the second part additionally shows the target and scan path positions and the flow fields predicted by the theoretical model for selected frames.

Targets are shown in green, laser scan paths in magenta with direction of flows indicated by blue arrows, and the flow lines of the theoretically predicted flows are shown in blue. The particles are made of fluorescent polystyrene with 1.63 and 2 µm diameter. Single images from the datasets shown here are shown in Fig. 4d.


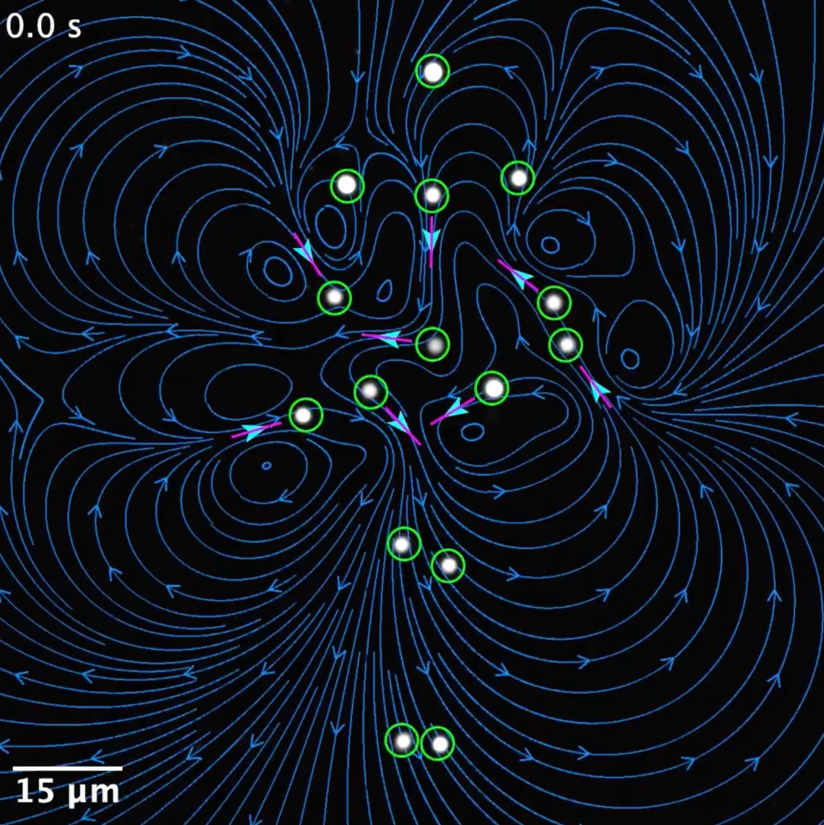


# Supplementary Video 9: Rapidly changing flow fields enabling the manipulation of the ‘female’ humanoid robot.

Targets are shown in green, laser scan paths in magenta with direction of flows indicated by blue arrows, and the flow lines of the theoretically predicted flows are shown in blue. The particles are made of fluorescent polystyrene with 1.63 and 2 µm diameter.


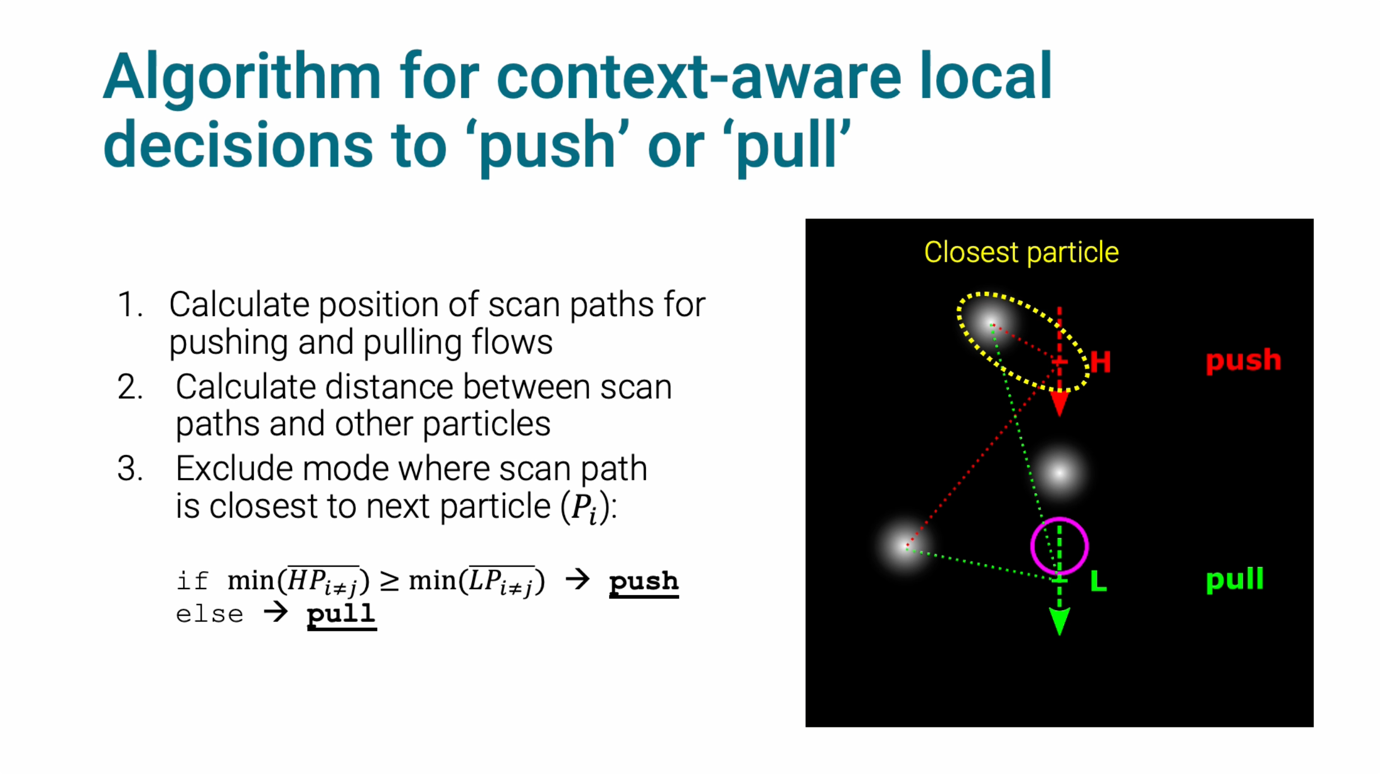


# Supplementary Video 10: Algorithm for context-aware local decisions to ‘push’ or ‘pull’.

This schematic video visualizes the algorithm for context-aware local decisions to ‘push’ or ‘pull’ based on the position of surrounding particles as described in detail in the methods section. In brief, the algorithm calculates the distance of the laser scan path to surrounding particles in the case of both push and pull and decides to use the mode where the closest particle is further away from the scan path.

# References

1. Tinevez, J.-Y. *et al.* TrackMate: An open and extensible platform for single-particle tracking. *Methods* **115**, 80–90 (2017).

2. Erben, E. *et al.* Feedback-based positioning and diffusion suppression of particles via optical control of thermoviscous flows. *Opt. Express* **29**, 30272–30283 (2021).

3. Stoev, I. D., Seelbinder, B., Erben, E., Maghelli, N. & Kreysing, M. Highly sensitive force measurements in an optically generated, harmonic hydrodynamic trap. *eLight* **1**, 7 (2021).

4. Favre-Bulle, I. A., Stilgoe, A. B., Scott, E. K. & Rubinsztein-Dunlop, H. Optical trapping in vivo: Theory, practice, and applications. *Nanophotonics* **8**, 1023–1040 (2019).

5. Català-Castro, F., Schäffer, E. & Krieg, M. Exploring cell and tissue mechanics with optical tweezers. *Journal of Cell Science* **135**, jcs259355 (2022).

6. Zhang, H. & Liu, K. K. Optical tweezers for single cells. *Journal of the Royal Society Interface* **5**, 671–690 (2008).

7. Neuman, K. C. & Nagy, A. Single-molecule force spectroscopy: Optical tweezers, magnetic tweezers and atomic force microscopy. *Nature Methods* **5**, 491–505 (2008).

8. Ramser, K. *et al.* Resonance Raman spectroscopy of optically trapped functional erythrocytes. *J. Biomed. Opt.* **9**, 593 (2004).

9. Bausch, A. R., Möller, W. & Sackmann, E. Measurement of local viscoelasticity and forces in living cells by magnetic tweezers. *Biophysical Journal* **76**, 573–579 (1999).

10. De Vlaminck, I. & Dekker, C. Recent advances in magnetic tweezers. *Annual Review of Biophysics* **41**, 453–472 (2012).

11. Cohen, A. E. & Moemer, W. E. Method for trapping and manipulating nanoscale objects in solution. *Applied Physics Letters* **86**, 1–3 (2005).

12. Cohen, A. E. Control of Nanoparticles with Arbitrary Two-Dimensional Force Fields. *Phys. Rev. Lett.* **94**, 118102 (2005).

13. Probst, R. & Shapiro, B. Three-dimensional electrokinetic tweezing: Device design, modeling, and control algorithms. *Journal of Micromechanics and Microengineering* **21**, 027004 (2011).

14. Yazbeck, R., Alibakhshi, M. A., Von Schoppe, J., Ekinci, K. L. & Duan, C. Characterization and manipulation of single nanoparticles using a nanopore-based electrokinetic tweezer. *Nanoscale* **11**, 22924–22931 (2019).

15. Li, H. *et al.* Precise electrokinetic position and three-dimensional orientation control of a nanowire bioprobe in solution. *Nat. Nanotechnol.* **18**, 1213–1221 (2023).

16. Ozcelik, A. *et al.* Acoustic tweezers for the life sciences. *Nature Methods* **15**, 1021–1028 (2018).

17. Meng, L. *et al.* Acoustic tweezers. *Journal of Physics D: Applied Physics* **52**, 273001 (2019).

18. Shi, J. *et al.* Acoustic tweezers: Patterning cells and microparticles using standing surface acoustic waves (SSAW). *Lab on a Chip* **9**, 2890 (2009).

19. Marzo, A. & Drinkwater, B. W. Holographic acoustic tweezers. *Proceedings of the National Academy of Sciences of the United States of America* **116**, 84–89 (2019).

20. Ding, X. *et al.* On-chip manipulation of single microparticles, cells, and organisms using surface acoustic waves. *Proc. Natl. Acad. Sci.* **109**, 11105–11109 (2012).

21. Lutz, B. R., Chen, J. & Schwartz, D. T. Hydrodynamic tweezers: 1. Noncontact trapping of single cells using steady streaming microeddies. *Analytical Chemistry* **78**, 5429–5435 (2006).

22. Schneider, T. M., Mandre, S. & Brenner, M. P. Algorithm for a microfluidic assembly line. *Physical Review Letters* **106**, 094503 (2011).

23. Tanyeri, M., Johnson-Chavarria, E. M. & Schroeder, C. M. Hydrodynamic trap for single particles and cells. *Applied Physics Letters* **96**, 224101 (2010).

24. Liu, X. *et al.* Hydrodynamic Tweezers: Trapping and Transportation in Microscale Using Vortex Induced by Oscillation of a Single Piezoelectric Actuator. *Sensors* **18**, 2002 (2018).

25. Qian, Y., Neale, S. L. & Marsh, J. H. Microparticle manipulation using laser-induced thermophoresis and thermal convection flow. *Scientific Reports* **10**, 19169 (2020).

26. Lin, L. *et al.* Thermophoretic Tweezers for Low-Power and Versatile Manipulation of Biological Cells. *ACS Nano* **11**, 3147–3154 (2017).

27. Lin, L., Hill, E. H., Peng, X. & Zheng, Y. Optothermal Manipulations of Colloidal Particles and Living Cells. *Accounts of Chemical Research* **51**, 1465–1474 (2018).

28. Wu, M. C. Optoelectronic tweezers. *Nature Photonics* **5**, 322–324 (2011).

29. Yang, S.-M. *et al.* Dynamic manipulation and patterning of microparticles and cells by using TiOPc-based optoelectronic dielectrophoresis. *Optics Letters* **35**, 1959 (2010).

30. Valley, J. K., Ningpei, S., Jamshidi, A., Hsu, H. Y. & Wu, M. C. A unified platform for optoelectrowetting and optoelectronic tweezers. *Lab on a Chip* **11**, 1292–1297 (2011).

31. Park, S. *et al.* Floating electrode optoelectronic tweezers: Light-driven dielectrophoretic droplet manipulation in electrically insulating oil medium. *Applied Physics Letters* **92**, 151101 (2008).

32. Wang, K., Schonbrun, E., Steinvurzel, P. & Crozier, K. B. Trapping and rotating nanoparticles using a plasmonic nano-tweezer with an integrated heat sink. *Nature Communications* **2**, 469 (2011).

33. Zhang, Y. *et al.* Plasmonic tweezers: for nanoscale optical trapping and beyond. *Light: Science and Applications* **10**, 59 (2021).

34. Ghorbanzadeh, M. & Darbari, S. Efficient Plasmonic 2D Arrangement and Manipulation System, Suitable for Controlling Particle–Particle Interactions. *Journal of Lightwave Technology* **37**, 2058–2064 (2019).

35. Zhang, Y. *et al.* Dynamic plasmonic nano-traps for single molecule surface-enhanced Raman scattering. *Nanoscale* **9**, 10694–10700 (2017).
